# A bioactive mammalian disaccharide associated with autoimmunity activates STING-TBK1-dependent immune response

**DOI:** 10.1038/s41467-019-10319-5

**Published:** 2019-05-30

**Authors:** Charles S. Fermaintt, Kanae Sano, Zhida Liu, Nozomi Ishii, Junichi Seino, Nicole Dobbs, Tadashi Suzuki, Yang-Xin Fu, Mark A. Lehrman, Ichiro Matsuo, Nan Yan

**Affiliations:** 1 Department of Immunology, University of Texas Southwestern Medical Center, Dallas, TX 75390 USA; 2 Division of Molecular Science, Gunma University, Maebashi, 371-8510 Japan; 3 Department of Pathology, University of Texas Southwestern Medical Center, Dallas, TX 75390 USA; 4 Glycometabolic Biochemistry Laboratory, RIKEN Cluster for Pioneering Research, Wako, 351-0198 Japan; 5 Department of Pharmacology, Department of Microbiology, University of Texas Southwestern Medical Center, Dallas, TX 75390 USA; 6 Department of Microbiology, University of Texas Southwestern Medical Center, Dallas, TX 75390 USA

**Keywords:** Glycobiology, Autoimmunity, Autoimmune diseases

## Abstract

Glycans from microbial pathogens are well known pathogen-associated molecular patterns that are recognized by the host immunity; however, little is known about whether and how mammalian self-glycans activate the host immune response, especially in the context of autoimmune disease. Using biochemical fractionation and two-dimensional HPLC, we identify an abundant and bioactive free glycan, the Manβ1-4GlcNAc disaccharide in *TREX1*-associated autoimmune diseases. We report that both monosaccharide residues and the β1-4 linkage are critical for bioactivity of this disaccharide. We also show that Manβ1-4GlcNAc is produced by oligosaccharyltransferase hydrolysis of lipid-linked oligosaccharides in the ER lumen, followed by ENGase and mannosidase processing in the cytosol and lysosomes. Furthermore, synthetic Manβ1-4GlcNAc disaccharide stimulates a broad immune response in vitro, which is in part dependent on the STING-TBK1 pathway, and enhances antibody response in vivo. Together, our data identify Manβ1-4GlcNAc as a novel innate immune modulator associated with chronic autoimmune diseases.

## Abstract (web summary)

Mammalian glycans have a role in host immunity but little is known about how they activate the host response in the context of autoimmune diseases. Here, the authors identify Manβ1-4GlcNAc as a novel innate immune modulator associated with chronic autoimmune diseases.

## Introduction

The seminal work of immunologist Karl Landsteiner around 1900 identifying ABO blood groups led to the discovery that glycans can be powerful immunogens in humans. Later work demonstrated that innate immunity often recognizes glycans on pathogens to prevent infection. The mammalian innate immune system has evolved pattern recognition receptors (PRRs) to recognize microbial components termed pathogen-associated pattern molecules (PAMPs), which range from nucleic acids to lipids, peptides, and glycans^1,2^. This immune surveillance network can also be erroneously activated by self-ligands that mimic microbial PAMPs, thus leading to chronic autoimmune diseases. One such example is *α-mannosidase-II* deficiency, which causes production of hybrid N-glycans that bear immune-stimulatory mannose-dependent ligands that promote autoimmune disease in mice similar to lupus^3^.

We recently characterized *TREX1*, a gene associated with several recessive and dominant autoimmune and autoinflammatory diseases^4^. TREX1 is an ER-anchored DNase with two independent functions, cytosolic DNA clearance function through the N-terminal DNase domain and free glycan anabolism regulation function through the C-terminal ER domain^5,6^. *Trex1*^*−/−*^ mice (loss of both DNase and glycan functions) develop severe early-onset systemic autoinflammatory disease with a short lifespan of 2–3 months^5,7^. In contrast, inactivating either DNase or glycan function alone in mice leads to less severe disease. For example, *TREX1-D18N* mutant mice disrupting only the DNase activity develop similar but significantly less severe disease phenotypes compared to *Trex1*^*−/−*^ mice, and these mutant mice also survive much longer^8^. We previously showed that *TREX1-V235fs* mutant mice express a DNase-active TREX1 truncation that lack glycan regulatory function and develop serologic autoimmunity by producing free glycans and autoantibodies against non-nuclear self-protein antigens^5,6^.

The glycan regulatory function of TREX1 is associated with its C-terminus. Frame-shift mutations that truncate TREX1 C-terminus are associated with dominant late-onset immune disorders, such as systemic lupus erythematosus (SLE) and retinal vasculopathy with cerebral leukodystrophy (RVCL)^9,10^. We previously demonstrated that loss of TREX1 C-terminus dysregulates the mammalian oligosaccharyltransferase (OST) activity leading to accumulation of free oligosaccharides (fOS) in the cell, and that fOSs activate interferon-stimulated genes (ISGs) in macrophages^5^. However, the identities of the bioactive fOSs and how they are sensed by the immune system remain elusive. Here, we describe a major bioactive mammalian fOS, Manβ1-4GlcNAc, from *Trex1*-associated autoimmune disease. We also define structural requirements for bioactivity and immune profile of this self-derived disaccharide as well as its biogenesis and immune sensing pathway.

## Results

### Identification of a bioactive mammalian disaccharide

**Fig. 1:**
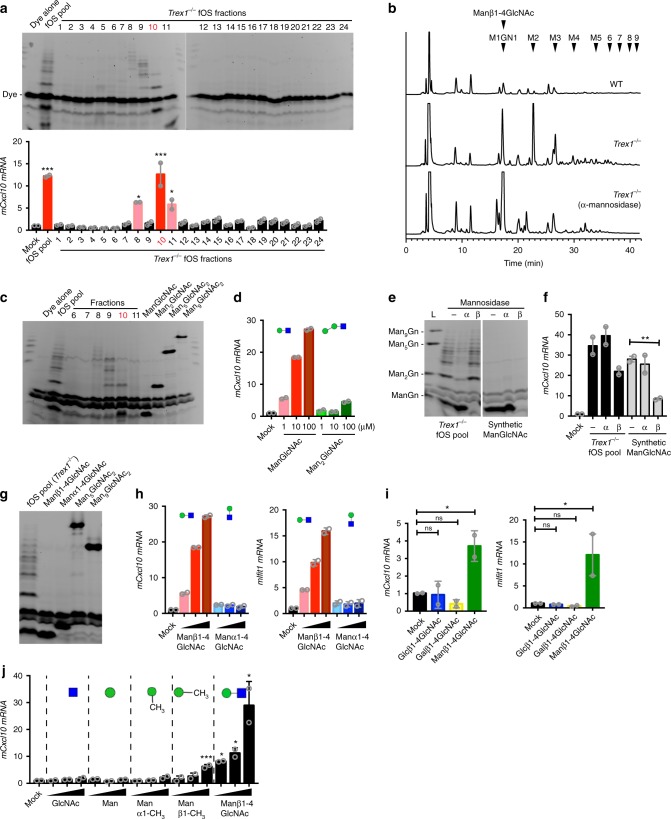
Identification of a bioactive mammalian disaccharide Manβ1-4GlcNAc. **a** Size exclusion fractionation of *Trex1*^*−/−*^ MEFs fOS pool and bioactivity of each fraction. Top panel, FACE analysis of each fraction. Bottom panel, quantitative RT-PCR analysis of *mCxcl10* mRNA in RAW264.7 cells (permeabilized by digitonin, same below) stimulated for 24 h with each fraction. **b** Two-dimensional HPLC analysis of fOS enriched in wild-type (WT), *Trex1*^*−/−*^ MEFs and *Trex1*^*−/−*^ fOS treated with α-mannosidases (see Methods). Quantitation and structure of top five enriched fOSs, identified by the second reverse-phase HPLC, are shown in Supplementary Fig. 2. **c** FACE analysis of *Trex1*^*−/−*^ MEFs fOS pool, key fractions and synthetic standards (as shown on top). **d** Quantitative RT-PCR analysis of *mCxcl10* mRNA in RAW264.7 cells that were stimulated with increasing amounts (1, 10, and 100 μM) of the synthetic Man_2_GlcNAc_1_ and ManGlcNAc_1_. **e, f** FACE analysis (**e**) and bioactivity (**f**) of untreated or α- or β-mannosidase digested *Trex1*^*−/−*^ MEFs fOS pool or the synthetic ManGlcNAc disaccharide. Bioactivity of each fOS sample was measured by quantitative RT-PCR analysis of *mCxcl10* mRNA in RAW264.7 cells stimulated for 24 h with indicated fOS samples. (**g**) FACE analysis of *Trex1*^*−/−*^ MEFs fOS pool, and synthetic Manβ1-4GlcNAc, Manα1-4GlcNAc, Man_9_GlcNAc_2_, Man_5_GlcNAc_2_. **h** Quantitative RT-PCR analysis of *mCxcl10* and *mIfit1* mRNA in RAW264.7 cells that were stimulated with increasing amounts (1, 10, and 100 μM) of the synthetic Manβ1-4GlcNAc and Manα1-4GlcNAc for 24 h. **i** Quantitative RT-PCR analysis of *mCxcl10* and *mIfit1* mRNA in RAW264.7 cells that were stimulated with 10 μM of Glcβ1-4GlcNAc, Galβ1-4GlcNAc, and Manβ1-4GlcNAc. **j** Quantitative RT-PCR analysis of *mCxcl10* mRNA in RAW264. 7 cells that were stimulated with increasing amounts (10, 100, and 1000 μM) of Mannose, GlcNAc, Manα1-CH_3_, Manβ1-CH_3_, and Manβ1-4GlcNAc. Data are representative of at least three independent experiments. Error bars indicate SEM. Unpaired *t*-test. *P < 0.05, **P < 0.01, ***P < 0.001,****P < 0.0001, ns not significant (same throughout)

We previously showed that fOS pools isolated from *Trex1*^*−/−*^ cells are immunogenic when incubated with macrophages^5^. To determine the specific glycan structure(s) that are responsible for immune activation, we performed size exclusion fractionation of the *Trex1*^*−/−*^ fOS pool and examined the bioactivity of each fraction on macrophages. We also analyzed each fraction by fluorophore-assisted carbohydrate electrophoresis (FACE). The majority of the fOS eluted in fractions #8-11 with larger structures eluting in fraction 8, medium structures in fraction 9, and smaller structures in fractions 10 and 11 (Fig. 1a). We then incubated fOS from each fraction as well as the non-fractioned fOS pool with RAW264.7 cells (a mouse macrophage cell line) for 24 h and measured immune activation. We chose *Cxcl10* mRNA expression as our initial ‘immune activity’ readout because it was the most induced ISG in *TREX1-V235fs* RVCL patient lymphoblast cells^5^. Fraction 10 stimulated *Cxcl10* the strongest; fraction 8 and 11 also appeared to be immunogenic but less potent compared to fraction 10 (Fig. 1a). The pattern of fOS fractionation and immune activity were highly consistent over four experiments. We also compared the immune profile of each fraction that contains fOS (#8-#11) by stimulating mouse bone marrow derived macrophages (BMDMs) and qRT-PCR array analysis of a panel of immune genes including type I interferon genes (IFN), IFN-stimulated genes (ISGs), inflammatory cytokine, and chemokine genes (Supplementary Fig. 1). We found that each fOS fraction stimulated a distinct immune profile. For example, fraction 10 stimulated the strongest *Cxcl10* expression, whereas fraction 9 stimulated the strongest *Il10* expression. Both fraction 10 and 11 stimulated *Ifn*a and *Cxcl2* expression to similar levels. These data suggest that multiple bioactive fOS structures exist in the *Trex1*^*−/−*^ fOS pool.

To gain detailed structural information on these fOSs, we also analyzed WT and *Trex1*^*−/−*^fOS pool by two-dimensional HPLC analysis (i.e., size-fractionation HPLC followed by dual-gradient reversed-phase HPLC^11^). The most enriched fOS structures in *Trex1*^*−/−*^ MEFs are paucimannose structures with one reducing-terminal GlcNAc (Man_1-3_GlcNAc) at ~20 fold higher levels than WT (Fig. 1b, Supplementary Fig. 2). Interestingly, the top three enriched glycan species match well with the predicted size of fOSs observed in fraction 10. We thus compared fraction 10 with chemically synthesized disaccharide (Manβ1-4GlcNAc, in-house, see Supplementary Methods), trisaccharide (Man_2_GlcNAc, in-house, see Supplementary Methods), and larger high-mannose glycans (Man_5_GlcNAc_2_ and Man_9_GlcNAc_2_, purchased from Sigma) by FACE. We found that the two major fOS species in fraction 10 co-migrate with the disaccharide (ManGlcNAc) and trisaccharide (Man_2_GlcNAc) standards (Fig. 1c). No other fractions contain detectable amount of disaccharide (ManGlcNAc) structure by FACE (Fig. 1c). Thus, we chose fraction 10 and the two fOS structures it contains for further analysis based on their high abundance and unique immune profile.

To see if any of these two fOS structures are bioactive, we stimulated RAW264.7 cells with increasing concentrations of the synthetic Man_2_GlcNAc and ManGlcNAc and measured *Cxcl10* mRNA expression. Only ManGlcNAc induced *Cxcl10* expression in a dose-dependent manner, while Man_2_GlcNAc lacked bioactivity in this assay (Fig. 1d). Together, these experiments suggest that the ManGlcNAc disaccharide is a bioactive glycan that is highly enriched in the *Trex1*^*−/−*^ fOS pool.

### Bioactivity is specific to only the Manβ1-4GlcNAc disaccharide

In our HPLC experiments we also analyzed *Trex1*^*−/−*^ fOS pool after digestion with α-mannosidases, and we found that the ManGlcNAc disaccharide peak was not affected by digestion, suggesting that the glycosidic bond is a β linkage (Fig. 1b). To determine if the β linkage is critical for the bioactivity of the ManGlcNAc disaccharide, we treated cellular *Trex1*^*−/−*^ fOS pool and the synthetic ManGlcNAc disaccharide with α- or β-mannosidase that selectively cleaves α- or β-mannosylated linkages, respectively, and assayed the digested glycan structures by FACE and bioactivity by stimulating macrophages and measuring *Cxcl10* expression. α-Mannosidase treatment removed most of the high molecular weight fOSs from the *Trex1*^*−/−*^ fOS pool but had no effect on the disaccharide band in the cellular fOS pool or synthetic ManGlcNAc disaccharide (Fig. 1e). In contrast, β-mannosidase digested the synthetic ManGlcNAc disaccharide as well as the disaccharide band in the fOS pool (Fig. 1e). β-mannosidase treatment reduced bioactivity of the fOS pool and bioactivity of the synthetic ManGlcNAc disaccharide, whereas α-mannosidase treatment had no effect (Fig. 1f). The residual activity in β-mannosidase-treated fOS pool indicates that other immunogenic fOS species also present in the fOS pool, consistent with our earlier observation that bioactivies were detected in multiple fractions from the fOS pool.

Glycosidic bonds can exist in two anomeric configurations, α or β. We next synthesized Manα1-4GlcNAc and compared its bioactivity to Manβ1-4GlcNAc **(**see Supplementary Methods). FACE analysis (which separates glycan based upon both oligosaccharide length and tertiary structural features that distinguish anomers and epimers) showed co-migration of Manβ1-4GlcNAc but not Manα1-4GlcNAc with the disaccharide band in the *Trex1*^*−/−*^ fOS pool (Fig. 1g). Remarkably, only Manβ1-4GlcNAc, but not Manα1-4GlcNAc, stimulated *Cxcl10* and *Ifit1* expression in RAW267.4 cells, suggesting that the bioactivity was associated with the β1-4 linkage (Fig. 1h). We next compared Manβ1-4GlcNAc to other disaccharides that also contain the β1-4 linkage, such as Glcβ1-4GlcNAc and Galβ1-4GlcNAc **(**see Supplementary Methods). Again, only Manβ1-4GlcNAc disaccharide was immunogenic (Fig. 1i). We also examined the bioactivity of monosaccharides such as Man, GlcNAc, α-methyl mannose (Manα1-CH_3_), and β-methyl mannose (Manβ1-CH_3_). None of the monosaccharides were able to stimulate *Cxcl10* expression to the extent of the Manβ1-4GlcNAc disaccharide in macrophages (Fig. 1j). Manβ1-CH_3_ had some activity but required 100 times higher concentration than Manβ1-4GlcNAc. We also analyzed fOS and immune profile of *TREX1-V235fs* mutant mouse BMDMs, and we found increased expression of *Cxcl10* and several other immune genes as well as significantly elevated levels of Manβ1-4GlcNAc disaccharide (Supplementary Fig. 3A, 3B). Together, these results identify Manβ1-4GlcNAc as an abundant and bioactive mammalian self-glycan in *TREX1*-associated autoimmune disease cells. Both the Man and the GlcNAc, as well as the β1-4 linkage, are important for full bioactivity of this disaccharide at micromolar concentrations.

### Biogenesis and cellular distribution of Manβ1-4GlcNAc

**Fig. 2:**
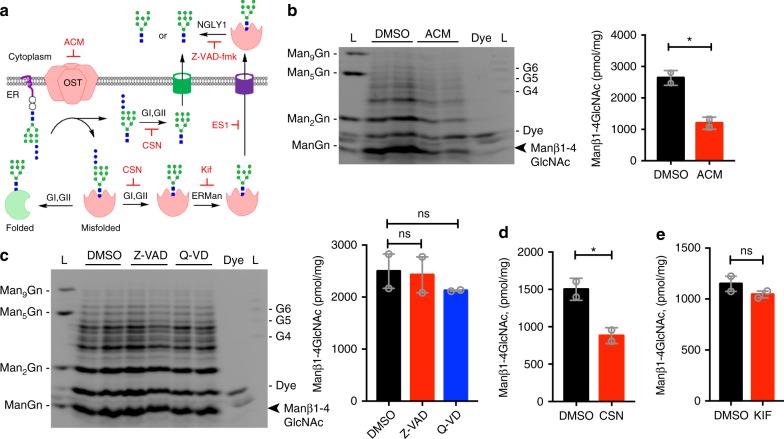
The Manβ1-4GlcNAc disaccharide originates from glycans produced by the OST. **a** A schematic diagram of two major biogenesis pathways of fOS in mammalian cells, OST hydrolysis of LLO and NGLY1 cleavage of N-glycans, and pharmacological inhibitors (red) that blocks either pathway. **b** FACE analysis of fOS pool isolated form *Trex1*^*−/−*^ MEFs treated with 1 μM aclacinomycin (ACM) for 24 h. Quantification of the Manβ1-4GlcNAc disaccharide band is shown on the right (same below). **c** FACE analysis of fOS pool isolated form *Trex1*^*−/−*^ MEFs treated with 30 μM Z-VAD or 50 μM Q-VD for 24 h. **d, e** Quantification of the Manβ1-4GlcNAc disaccharide in *Trex1*^*−/−*^ MEFs treated with 100 μM of castanospermine (CSN, D) or 100 μM of kifunensine (KIF, E) for 24 h. Representative FACE gels are in Supplementary Fig. 4. Data are representative of at least three independent experiments. Error bars indicate SEM. Unpaired *t*-test

Oligomannose fOS can be generated from deglycosylation of ER-associated degradation (ERAD) substrates by N-glycanase NGLY1 or from the hydrolysis of lipid-linked oligosaccharide (LLOs) by the oligosaccharyltransferase (OST)^12,13^. We previously showed that absence of TREX1 or its C-terminus (e.g., *TREX1-V235fs* mutant) dysregulates the OST activity leading to accumulation of intracellular fOS^5^. To determine the source that leads to production of the Manβ1-4GlcNAc disaccharide, we used specific inhibitors that block either ERAD or OST activity and measured their effect on fOS production in *Trex1*^*−/−*^ MEFs by FACE (Fig. 2a diagram). Inhibiting OST activity with aclacinomycin A (ACM^5,14^) led to significant reduction of Manβ1-4GlcNAc disaccharide as well as other high-mannose fOSs (Fig. 2b). In contrast, inhibiting of NGLY1 with Z-VAD-fmk^15^ had no effect on the Manβ1-4GlcNAc disaccharide or any other fOSs (Fig. 2c). We next examined ER translocation and glycan processing machinery in the lumen. Inhibiting ER glucosidases with castanospermine (CSN) blocks both the translocation of fOS to the cytoplasm as well as the progress through ERAD^16,17^, while inhibition of ER lumenal mannosidases with kifunensine (KIF) only affects misfolded protein tagging for ERAD^18,19^. *Trex1*^*−/−*^ MEFs treated with CSN significantly reduced Manβ1-4GlcNAc disaccharide while treatment with KIF had no impact, suggesting that fOS derived from LLO hydrolysis are exported to the cytosol as large structures before trimming by ER luminal mannosidases (Fig. 2d, e and Supplementary Fig. 4A, 4B). We also tested cycloheximide (CHX, inhibits protein synthesis), MG132 (inhibits proteasomal degradation), and eeyarestatin I (ES1, inhibits ERAD), and none had any effect on the Manβ1-4GlcNAc disaccharide level in *Trex1*^*−/−*^ MEFs (Supplementary Fig. 4C-4K). These data suggest that OST-hydrolyzed LLOs are precursors of the Manβ1-4GlcNAc disaccharide.

**Fig. 3:**
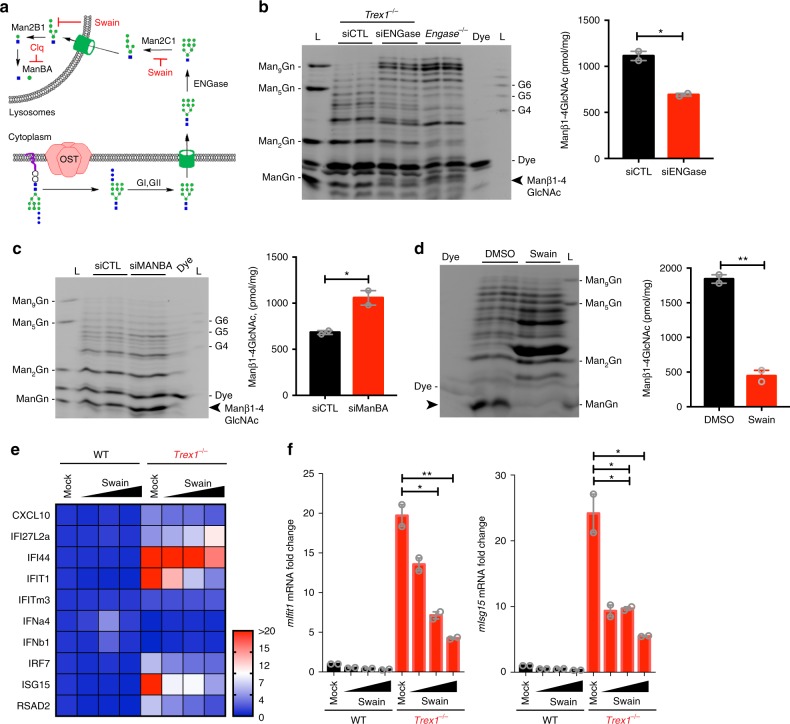
The Manβ1-4GlcNAc disaccharide biogenesis requires processing by ENGase and α-mannosidase. **a** A schematic diagram of the catabolic processing of luminal fOS and pharmacological inhibitors (red) that blocks each step. **b** FACE analysis of fOS pool isolated from *Trex1*^*−/−*^ MEFs treated with si-control or si-ENGase for 48 h or fOS pool isolated from untreated *Engase*^*−/−*^ MEFs. Quantification of the Manβ1-4GlcNAc disaccharide is shown on the right (same below). **c** FACE analysis of fOS pool isolated from *Trex1*^*−/−*^ MEFs treated with si-control or si-ENGase for 48 h. Similar to B. **d** FACE analysis of fOS pool isolated from *Trex1*^*−/−*^ MEFs treated with 10 μM Swain for 24 h. **e**, **f** Quantitative RT-PCR array analysis of immune gene expression in WT and *Trex1*^*−/−*^ E15.5 primary MEFs treated with mock or Swain (0.1, 1, and 10 μM) for 24 h. A heat map summarizing multiple immune genes is shown in **e** and two representative ISGs are shown in **f**. Data are representative of at least three independent experiments. Error bars indicate SEM. Unpaired *t*-test

We next investigated how fOSs generated from LLOs get further processed into the Manβ1-4GlcNAc disaccharide. fOS derived from N-glycosylated proteins are processed by endo-β-N-acetylglucosaminidase (ENGase) in the cytosol. We thus examined the role of the ENGase pathway, which includes first trimming fOSs to Man_5_GlcNAc, transfer into the lysosomes and then further processed by α- and β-mannosidases into Manβ1-4GlcNAc disaccharide and monosaccharides^20–22^ (Fig. 3a diagram). ENGase knockdown by siRNA in *Trex1*^*−/−*^ MEFs led to reduced Manβ1-4GlcNAc disaccharide levels as well as a corresponding increase in high molecular weight fOSs that co-migrated with previously characterized species in *Engase*^*−/−*^ MEFs^23^ (Fig. 3b and Supplementary Fig. 5A). Conversely, *MANBA* (encodes β-mannosidase) knockdown led to increased Manβ1-4GlcNAc disaccharide level, further confirming the β linkage in the disaccharide and suggesting that β-mannosidase is the critical last enzyme in the pathway that breaks down the disaccharide (Fig. 3c and Supplementary Fig. 5B). Chloroquine treatment (CQ, lysosome neutralizer, thus inactivates β-mannosidase in the lysosome) also increased the disaccharide (Supplementary Fig. 5C, 5D). Because mammalian cells have multiple genes encoding α-mannosidase, we next treated *Trex1*^*−/−*^ MEFs with a broad α-mannosidase inhibitor swainsonine (Swain^24^). Swainsonine completely eliminated the Manβ1-4GlcNAc disaccharide from *Trex1*^*−/−*^ fOS and accumulated the Man_3-5_GlcNAc intermediates (Fig. 3d). We previously showed that ACM treatment inhibits OST activity and reduces ISG expression in *Trex1*^*−/−*^ cells^5^. We next treated WT and *Trex1*^*−/−*^ MEFs with increasing dose of swainsonine, and we observed dose-dependent decrease of ISG expression (Fig. 3e, 3f). These data suggest that LLO-derived fOSs move from the ER lumen to the cytosol followed by processing by ENGase and α-mannosidases to produce the Manβ1-4GlcNAc disaccharide. Also, interruption of Manβ1-4GlcNAc biogenesis in *Trex1*^*−/−*^ cells reduces immune activation.

We next wanted to examine the cellular distribution of the mammalian Manβ1-4GlcNAc disaccharide. Treating the *Trex1*^*−/−*^ MEFs with digitonin (10 μg/mL) that only permeabilized the plasma membrane^25^ led to lost of nearly half of the disaccharide, suggesting that a substantial fraction of Manβ1-4GlcNAc resides in the cytosol (Supplementary Fig. 5E). We also compared fOSs isolated from media and from cell lysates originated from the same tissue culture dish with a monolayer of healthy cells, and we found similar amount of the Manβ1-4GlcNAc disaccharide in both intracellular and extracellular compartments (Supplementary Fig. 5F). Together, these findings demonstrate that the bioactive Manβ1-4GlcNAc disaccharide resides in both intracellular and extracellular space.

### Manβ1-4GlcNAc activates immune signaling via TBK1 and NF-κB

**Fig. 4:**
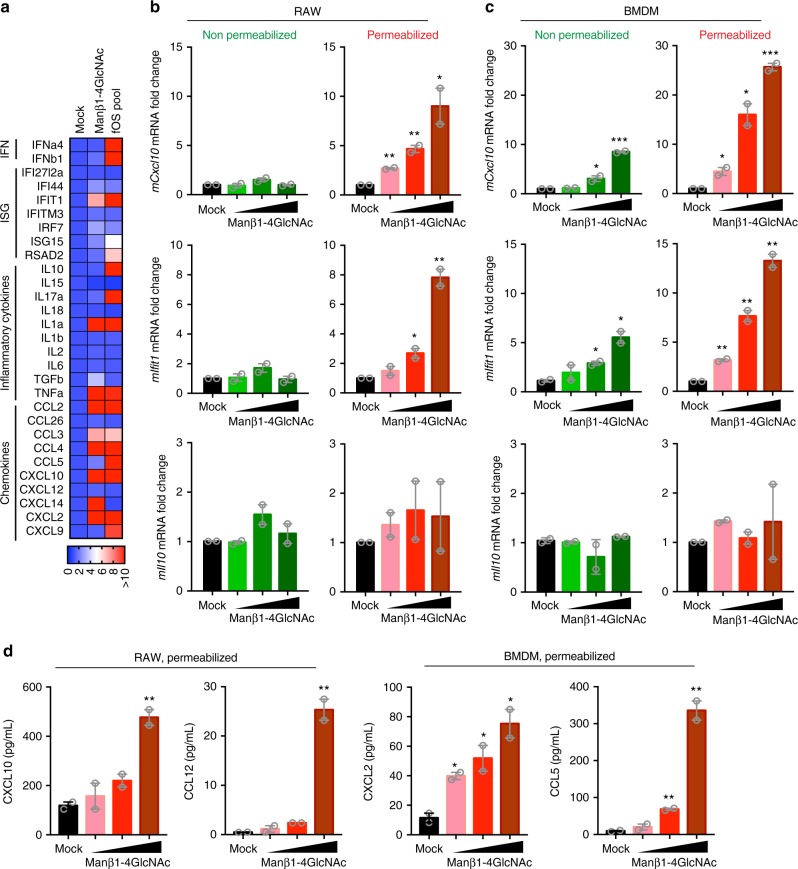
The Manβ1-4GlcNAc disaccharide activates an intracellular pathway. **a** A heat map showing immune gene expression profiles induced by *Trex1*^*−/−*^ fOS pool or the Manβ1-4GlcNAc disaccharide. Permeabilized RAW264.7 cells were stimulated with 10 μM of the *Trex1*^*−/−*^ fOS pool or the synthetic Manβ1-4GlcNAc disaccharide followed by quantitative RT-PCR analysis of each indicated mRNA. **b**, **c** Quantitative RT-PCR analysis of *mCxcl10*, *mIfit1 and mIl10* mRNA in RAW264.7 cells (**b**) or BMDMs (**c**) that were either non-permeabilized or permeabilized by digitonin (as indicated on top), then cells were treated with mock or increasing amounts (1, 10 and 100 μM) of the Manβ1-4GlcNAc disaccharide for 24 h. **d** Multiplex ELISA analysis of cytokines and chemokines from experiments in **b** and **c**. Data are representative of at least two independent experiments. Error bars indicate SEM. Unpaired *t*-test

We performed RNA-seq to investigate the immune response activated by the synthetic Manβ1-4GlcNAc disaccharide or cellular *Trex1*^*−/−*^ fOS pool in RAW267.4 macrophages. We found that 2044 genes were differentially expressed after the Manβ1-4GlcNAc disaccharide stimulation, 1131 genes after fOS pool stimulation, and 760 genes are shared between the two (Supplementary Fig. 6A). Ingenuity pathways analysis on the 760 shared genes revealed top pathways involved in T helper cell differentiation, antigen presentation, and upregulation of various chemokine genes from the CXCL family (Supplementary Fig. 6B). We validated several groups of immune genes including IFN, ISGs, inflammatory genes, and chemokine genes by qRT-PCR. The Manβ1-4GlcNAc disaccharide and fOS pool stimulated broad expression of immune genes including IFN and ISGs (as we observed before^5^) and many CXCL and CCL family chemokine genes (Fig. 4a).

To investigate whether Manβ1-4GlcNAc is detected by intracellular or extracellular receptors, we prepared permeabilized and non-permeabilized RAW267.4 cells and BMDMs and incubated them with Manβ1-4GlcNAc disaccharide. Primary macrophages such as BMDMs are more efficient at phagocytosis than macrophage cell lines such as RAW267.4 cells^26^. We found that Manβ1-4GlcNAc disaccharide only stimulated immune gene expression (e.g., *Cxcl10*, *Ifit1*) in permeabilized RAW267.4 cells and not in non-permeabilized RAW267.4 cells (Fig. 4b). In contrast, both permeabilized and non-permeabilized BMDMs showed dose-dependent and significant increase of immune gene expression after incubation with Manβ1-4GlcNAc disaccharide in the media, with permeabilizated BMDMs showing more robust response (Fig. 4c). Manβ1-4GlcNAc also induced increased cytokine and chemokine secretion in the media in permeabilized cells (Fig. 4d). Manβ1-4GlcNAc did not induce IL-10 expression in any of the conditions (Fig. 4b, c). These data suggest the possibility of an intracellular sensing pathway for Manβ1-4GlcNAc in macrophages.

**Fig. 5:**
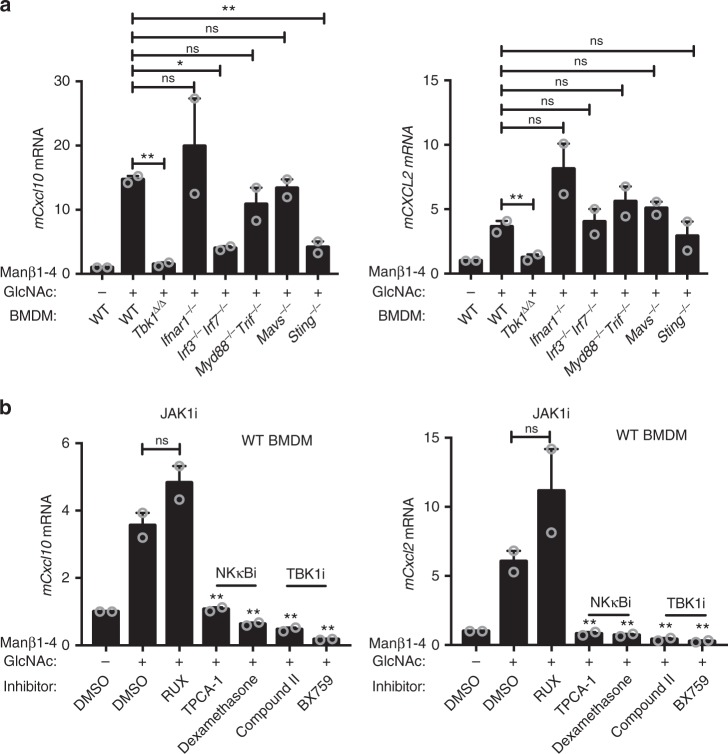
The Manβ1-4GlcNAc disaccharide activates TBK1- and NF-κB-dependent immune response. **a** Quantitative RT-PCR analysis of *mCxcl10* and *mCxcl2* mRNA in knockout BMDMs (as indicated on the bottom) that were treated with 10 μM of the Manβ1-4GlcNAc disaccharide for 24 h. **b** Quantitative RT-PCR analysis of *mCxcl10* and *mCxcl2* mRNA in BMDMs that were pretreated with the indicated inhibitors for 1 h and then treated with 10 μM of the Manβ1-4GlcNAc disaccharide for 24 h. RUX, rituximab (JAK1/2 inhibitor), TPCA-1 and Dexamethasone are NF-κB inhibitors, Compound II and BX759 are TBK1 inhibitors. See Supplementary Fig. 6 for a diagram showing innate immune pathways tested here. Data are representative of at least three independent experiments. Error bars indicate SEM. Unpaired *t*-test

We next sought to determine which immune pathway is required for sensing the Manβ1-4GlcNAc disaccharide. We chose a panel of knockout mice that are deficient in genes required for key innate immune signaling pathways (see Supplementary Fig. 6C for a diagram). TBK1 is a protein kinase required by several intracellular innate immune signaling pathways. IFN receptor 1 (IFNAR1) is required for type I IFN response. IRF3 and IRF7 are key transcription factors for IFN and ISG mRNA expression. MYD88 and TRIF are the two essential adaptor proteins required for TLR signaling. MAVS and STING are key non-redundant adaptor proteins for cytosolic RNA and DNA sensing pathways, respectively. We compared the response to the Manβ1-4GlcNAc disaccharide on BMDMs from wild-type, *Tbk1*^*Δ/Δ*^, *Ifnar1*^*−/−*^, *Irf3*^*−/−*^*Irf7*^*−/−*^, *Myd88*^*−/−*^*Trif*^*−/−*^, *Mavs* ^*−/−*^, and *Sting* ^*−/−*^ mice. We found that wild-type, *Ifnar1*^*−/−*^, *Myd88*^*−/−*^*Trif*^*−/−*^ and *Mavs−/−* BMDMs responded to the Manβ1-4GlcNAc disaccharide stimulation to similar levels. In contrast, *Tbk1*^*Δ/Δ*^ BMDMs completely ablated immune response to Manβ1-4GlcNAc while *Sting*^*−/−*^ and *Irf3*^*−/−*^*Irf7*^*−/−*^ partially suppressed immune activation (Fig. 5a). To corroborate and extend on these findings, we pretreated wild-type BMDMs with TBK1 inhibitors (BX795 and Compound II), NF-κB inhibitors (TPCA-1 and dexamethasone), and JAK1/2 inhibitor ruxolitinib (inhibits type I IFN signaling), then stimulated with the Manβ1-4GlcNAc disaccharide. TBK1 and NF-κB inhibitors suppressed the activation of *Cxcl10* and *Cxcl2* while ruxolitinib had no effect (Fig. 5b). Taken together, these studies suggest that Manβ1-4GlcNAc-activated immune response is TBK1- and NF-κB-dependent but IFN- and TLR-independent.

### Immune gene profile activated by Manβ1-4GlcNAc

**Fig. 6:**
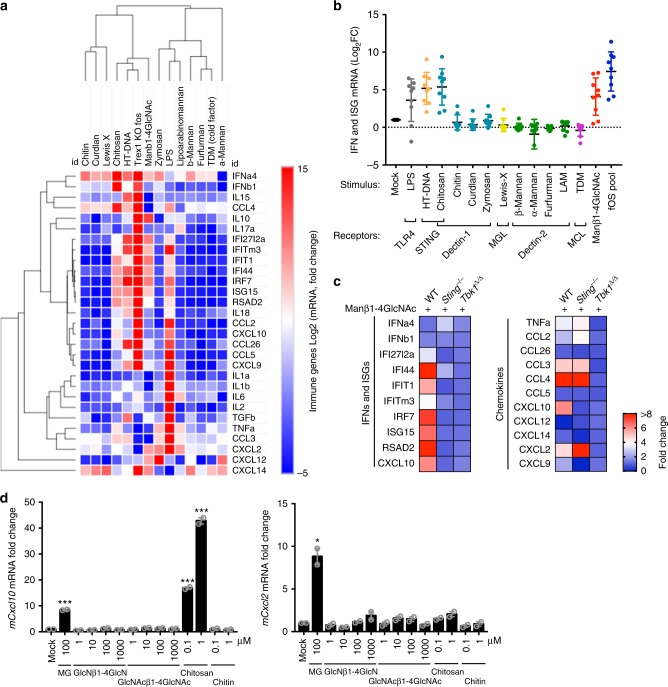
The Manβ1-4GlcNAc disaccharide activates a broad immune gene signature that is similar to those acticated by DNA or chitosan. **a** Hierarchical heat map analysis of immune gene (as indicated on the right) expression in BMDMs treated with various ligands (as indicated on top) for 24 h. mRNA expression of each gene was measured by qRT-PCR. **b** IFN and ISGs immune gene expression in BMDMs treated with various ligands (as indicated on bottom). Same gene expression data set as in **a**. Each dot represents one gene. **c** A heat map of immune gene expression in WT, *Tbk1*^*Δ/Δ*^ and *Sting*^−/−^ BMDMs stimulated with Manβ1-4GlcNAc disaccharide for 24 h. mRNA expression of each gene was measured by qRT-PCR. **d** Quantitative RT-PCR analysis of *mCxcl10* and *mCxcl2* mRNA in BMDMs that were treated with mock or indicated glycans (bottom) for 24 h. mRNA expression of each gene was measured by qRT-PCR. Data are from a representative set of at least two independent experiments. Error bars indicate SEM. Unpaired *t*-test

Myeloid cells, which include macrophages, express an arsenal of carbohydrate binding proteins known as C-type lectins receptors (CLR) that function like PRRs and recognize a wide variety of glycan PAMPs. There are hundreds of lectins that can be potential candidate receptors for Manβ1-4GlcNAc disaccharide, making the individual knockdown approach impractical. It is also possible that Manβ1-4GlcNAc disaccharide represents a distinct type of bioactivity with its unique monovalent structure and micromolar activity concentration. Thus, we examined whether the Manβ1-4GlcNAc disaccharide stimulates an immune profile that can be matched to other non-mammalian bioactive glycans or non-glycan agonists. We stimulated BMDMs with Dectin-1 ligands (Chitosan, Chitin, Curdlan, and Zymosan), MGL ligand (Lewis-X)^6^, Dectin-2 ligands (β-Mannan, α-Mannan, Furfurman, and Lipoarabinomannan), and MCL/MINCLE ligand (Cord factor) as well as HT-DNA (herring testis DNA, activates cytosolic cGAS-STING-TBK innate immune pathway), LPS (activates TLR4 pathway), Manβ1-4GlcNAc disaccharide, and fOS pool. We then measured the expression of IFN, ISGs, inflammatory, and chemokine genes by qRT-PCR array. After hierarchical clustering that would group ligands with similar immune profiles, Manβ1-4GlcNAc disaccharide and the fOS pool emerged most similar to HT-DNA and chitosan (Figs. 6a, b). Both HT-DNA and chitosan activate the STING-TBK1 pathway in the cytosol^27^. We next analyzed the STING-TBK1 pathway in more detail by comparing a broader panel of immune genes activated by Manβ1-4GlcNAc disaccharide in WT, *Tbk1*^*Δ/Δ*^, and *Sting*^−/−^ BMDMs. *Tbk1*^*Δ/Δ*^ ablated all examined immune gene activation by Manβ1-4GlcNAc. Interestingly, *Sting*^−/−^ eliminated expression of ISGs (e.g., *Ifi44, Ifit1, Isg15*, etc) but had little effect on chemokine genes (e.g., *Ccl2*, *Ccl3*, *Cxcl2*, etc. Note: Cxcl10 is both an ISG and a chemokine) in response to Manβ1-4GlcNAc (Fig. 6c).

Considering the similarities in immune profile between Manβ1-4GlcNAc and chitosan, we next compared two additional disaccharides, N,N′-diacetylchitobiose (GlcNAcβ1-4GlcNAc) and chitobiose (GlcNβ1-4GlcN), as well as their respective polymers, chitin and chitosan. Neither disaccharide structure stimulated *Cxcl10* or *Cxcl2* expression in BMDM at up to 1000 μM, while Manβ1-4GlcNAc stimulated significant increased immune response at 100 μM. Chitosan, but not chitin, also stimulated robust *Cxcl10* expression in BMDMs at very low concentration (0.1 μM). These data further support the unique structure and immune activity of Manβ1-4GlcNAc in comparison to other disaccharides. It also raises the possibility that polymeric form of Manβ1-4GlcNAc (e.g., similar to chitobiose versus chitosan) could have more potent immune activities.

### Manβ1-4GlcNAc enhances antibody response in vivo

**Fig. 7:**
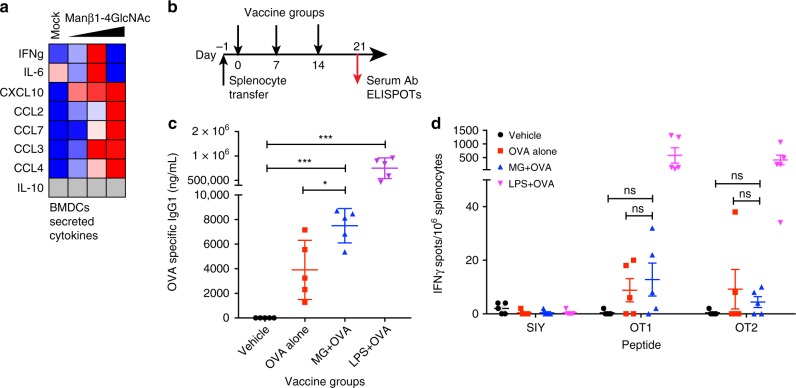
The Manβ1-4GlcNAc disaccharide enhances antibody response in vivo. **a** Multiplex ELISA analysis of cytokines and chemokines secreted by BMDCs treated with mock or increasing amounts (1, 10, and 100 μM) of the Manβ1-4GlcNAc disaccharide for 24 h. **b** A schematic diagram of Manβ1-4GlcNAc mouse immunization experiment. See Method for more details. **c** OVA-specific IgG1 antibody titre by ELISA using serum at day 21. Four vaccination groups are indicated on the bottom. MG, Manβ1-4GlcNAc. *N* = 5. **d** IFNγ ELISPOT assay using splenocytes at day 21. Five μg/ml of OT1 OVA (257-264), OT2 OVA (323-339), or SIY (SIYRYYGL) peptide (negative control) were used to re-stimulate the antigen specific T cells. IFN-γ production was determined 48 h later. Error bars indicate SEM. Unpaired *t*-test

To further substantiate our findings, we stimulated BMDCs with Manβ1-4GlcNAc and found increased secretion of multiple chemokines, including Cxcl10, Ccl2, Ccl3 (Fig. 7a). We did not detect activation markers of BMDCs such as MHC-II, CD86, CD80 after Manβ1-4GlcNAc stimulation (Supplementary Fig. 7A, B). We also stimulated wild-type splenocytes ex vivo with the disaccharide and did not observe direct activation of either T or B cells (Supplementary Fig. 7C). All glycans are chemically synthesized and confirmed by structural analysis (Supplementary Figs. 8–20). We next tested the activity of Manβ1-4GlcNAc in vivo. We first transferred mixed OT1 and OT2 splenocytes into wild-type mice, then immunized subcutaneously with either vehicle, OVA alone, OVA + MG (Manβ1-4GlcNAc) or OVA + LPS on day 0, 7, and 14. Seven days after the last immunization, we collected serum for antibody analysis and splenocytes for IFNγ ELISPOT assay. We detected significantly higher OVA-specific IgG1 in OVA + MG-treated mice compared to those treated with OVA alone, suggesting that Manβ1-4GlcNAc enhanced B cell-mediated antibody response in vivo (Fig. 7b). Manβ1-4GlcNAc did not significantly enhance T cell response (Fig. 7b). Interestingly, we previously showed that *TREX1-V235fs* mice develop serologic autoimmunity with increased autoantibody production in the serum and enhanced immune gene expression in cells^6^. Thus, together, our in vitro and in vivo data suggest that Manβ1-4GlcNAc activates a broad immune profile in DCs and macrophages that are mediated in part through STING and other immune sensing pathway(s) that converge on TBK1, which lead to enhanced antibody response in vivo.

## Discussion

Mammalian self-nucleic acids are well known for their causal association with autoimmune disease. Much less is known about whether and how mammalian self-glycans also activate immune responses associated with autoimmunity. Here, we identified a bioactive fOS, the Manβ1-4GlcNAc disaccharide, that is produced by OST hydrolysis of LLOs in the ER lumen, processed by ENGase and mannosidases in the cytosol, and sensed in part by the cytosolic STING-TBK1-dependent innate immune pathway.

We found that the bioactivity of Manβ1-4GlcNAc disaccharide requires both monosaccharides as well as the β1-4 linkage, and this disaccharide is surprisingly active in comparison to other disaccharide we tested. The immune profile of Manβ1-4GlcNAc is remarkably similar to that of chitosan, which also activates the STING-TBK1 pathway^27^. Structurally distinct chitosans also promote IFN or inflammasome responses^28^. Interestingly, chitosan is a polysaccharide of β1-4-linked glucosamine (GlcN), and we found that GlcNβ1-4GlcN disaccharide alone does not have detectable immune activities even at high concentrations. Thus, given the impressive bioactivity of Manβ1-4GlcNAc, we expect that a polysaccharide containing repeats of Manβ1-4GlcNAc could potentially have very potent immune-stimulating activities.

We further defined the immunogenicity of the Manβ1-4GlcNAc disaccharide in vivo and in vitro. Using an immunization experiment with the model antigen OVA, we found that the disaccharide stimulated significantly increased antibody response but not T cell response, although the overall response is relatively weak. This is not surprising because: (1) The disaccharide is only one of several immunogenic glycans we identified; (2) Disaccharides are not expected to be highly immunogenic, and polysaccharides are. (3) The receptor for Manβ1-4GlcNAc is likely intracellular. Nonetheless, the enhanced antibody response is consistent with our previous findings that *TREX1-V235fs* mice develop serologic autoimmunity^6^. We also defined the cell subsets affected by Manβ1-4GlcNAc. Myeloid cells such as macrophages are directly activated by Manβ1-4GlcNAc. DCs do respond to Manβ1-4GlcNAc although to a less extent comparing to macrophages. Manβ1-4GlcNAc does not directly activate T and B cells when stimulated ex vivo. Thus, it is likely that Manβ1-4GlcNAc primarily affects myeloid cells such as macrophages (through intracellular receptors) and induces expression of inflammatory cytokines and chemokines that promote a predominant B cell-mediated antibody response. Future studies depleting these myeloid populations will help define their function in vivo.

Although the Manβ1-4GlcNAc disaccharide has not been found on pathogens as a PAMP, it has been associated with microbial metabolic activity for colonization or infection^29,30^. Bacterial pathogens digest mammalian N-glycans on cells to facilitate infection. In doing so, many bacterial pathogens produce the Manβ1-4GlcNAc disaccharide and other fOSs, which are typically considered as by-products^31^. Therefore, it is possible that these mammalian fOSs produced during infection could function as danger-associated molecular patterns (DAMPs) that alert the host of the infection. In support of this notion, our previous work has demonstrated that fOSs are produced when cells are undergoing ER stress or infected with herpes simplex virus 1 (HSV-1)^32^.

Defects in cellular glycan processing enzymes can lead to chronic immune disorders. *α-mannosidase-II* deficiency leads to production of N-glycans that bear immune-stimulatory mannose-dependent ligands that promote autoimmune diseases similar to lupus^3^. Deficiency of *β-mannosidase* in humans and mice cause a chronic disease called β-mannosidosis, which causes accumulation of the Manβ1-4GlcNAc disaccharide in multiple disease-affected tissues^33,34^ and neuropathological characteristics that resemble inflammation^35,36^. We previously showed that *Trex1*-deficiency or *fs* mutations dysregulate the OST complex leading to production of immunogenic high-mannose fOS^5,6^. Our data here further show that multiple bioactive fOS species are produced when OST is dysregualted, potentially each with distinct immune profile. Thus, our data reveal an exciting possibility for distinct mammalian fOSs, such as Manβ1-4GlcNAc defined here, to play a role in autoimmune disease pathogenesis. Lastly, our inhibitor studies targeting the biogenesis or immune sensing pathways of Manβ1-4GlcNAc demonstrated several avenues of therapeutics for treating *TREX1-fs* RVCL, β-mannosidosis, and potentially other chronic autoimmune diseases.

## Methods

### Cells, antibodies, and reagents

MEFs, RAW264.7, BMDM, and BMDC were maintained in Dulbecco’s modified Eagle’s medium (DMEM) with 10% (v/v) heat-inactivated fetal bovine serum (FBS), with 2 mM L-glutamine, 10 mM HEPES, and 1 mM sodium pyruvate (complete DMEM) with the addition of 100 U/ml penicillin, 100 mg/ml streptomycin except MEFs and cultured at 37 °C with 5% CO_2_. MEFs, BMDM, and BMDCs were generated from WT C57BL/6 mice^37^. RAW264.7 cells were obtained from ATCC. Antibodies used in this study include anti-ubiquitin (rabbit, 1:1000 dilution, P4D1, Cell Signaling Technology), anti-Tubulin (mouse, 1:20,000 dilution, B-5-1-2, Sigma–Aldrich), anti-LC3 (rabbit, 1:1000 dilution,ab48394, Abcam), anti-STING (rabbit, 1:500 dilution, D2P2F, Cell Signaling), anti-GAPDH (rabbit, 1:1000 dilution, sc-25778, Santa Cruz), anti-Rab5 (rabbit, 1:1000 dilution,ab109534, Abcam), and anti-HMGB1 (rabbit; 1:2,000 dilution;ab18256; Abcam). The inhibitors used in this study are ACM (1 uM, Santa Cruz), Z-VAD-fmk (30 uM, BD), Q-VD-OPh (50 uM, BD), CSN (100 uM, Sigma–Aldrich), Kif (100 uM, Sigma–Aldrich), ES1 (10 uM, Sigma–Aldrich), CHX (10 ug/ml, Sigma–Aldrich), MG132 (1 uM, Sigma–Aldrich), Swain (10 uM, Tocris), Clq (10 uM, Sigma–Aldrich), Compound II (1 uM, UTSW), BX759 (1 uM, Invivogen), Ruxolitinib (1 uM, Invivogen), Dexamethasone (100 nM, Invivogen), and TPCA1 (1 uM, Sigma–Aldrich). The ligands used to stimulate macrophages are GalGlcNAc (Sigma–Aldrich), Mannose (Sigma–Aldrich), GlcNAc (Sigma–Aldrich), Manα1-CH_3_ (Sigma–Aldrich), Manβ1-CH_3_(Santa Cruz), LPS (100 ng/ml, Invivogen), htDNA (1 ug, Sigma–Aldrich), Chitosan (10 ug/ml, Invivogen), Chitin (10 ug/ml, Sigma–Aldrich), Curdlan (10 ug/ml, Invivogen), Zymosan (10 ug/ml, Invivogen), Le^X^ (10 ug/ml,Sigma–Aldrich), β-Mannan (10 ug/ml, Megazyme), α-Mannan (10 ug/ml, Sigma–Aldrich), Furfurman (10 ug/ml, Invivogen), Lipoarabinomannan (1 ug/ml, Invivogen), and Cord factor (1 ug/ml, Invivogen). The alpha-mannosidase used in Fig. 1b is alpha(1-2,3,6) mannosidase from Jack bean (Prozyme, catalog number GKX-5010). It can remove all alpha-mannose from high-mannose-type glycans (unless they are capped by glucose). All uncropped western blot scans are in Source Data.

### Glycan analysis

Fluorophore-assisted carbohydrate electrophoresis (FACE) analysis was done as follows (also described in ref. ^5^). Briefly, MEFs were plated at 75–80% confluency and transfected (RNAiMAX, Thermo Fisher) with the ENGase siRNA (Sigma–Aldrich) or treated with inhibitors for 24 h and disrupted with methanol the next day. The methanol disrupted cells or the lyophilized media were subject to a three-phase (aqueous, interface, and organic) extraction, which yields an aqueous fraction containing neutral fOS (desalted by ion exchange chromatography) and an interface fractions, which was extracted with chloroform:methanol:water (10:10:3) to isolate total proteins that was used to normalize the loading of labeled fOS to gels, immunoblotting and for the isolation of N-linked glycans by PNGase F digestion (New England Biolabs). Isolated glycans (fOS and digested N-glycans) were conjugated with 7-amino-1,3-naphthalenedisulfonic acid (ANDS, AnaSpec) and subjected to reductive amination with NaBH_3_CN (Sigma–Aldrich) for 24 h at 37 °C. Labeled fOS and digested N-glycans were resolved on an oligosaccharide profiling gel, with 10 pmol of glucose oligomers (ranging from four to seven glucosyl residues) and 100 pmol of Man_9_GlcNAc_2_ (Sigma–Aldrich), Man_5_GlcNAc_2_ (Sigma–Aldrich), Man_2_GlcNAc and ManGlcNAc as standards. Gels were visualized using a UVP Chemidoc-ItII scanner and quantified with VisionWorks software.

### RNA isolation, RNA sequencing, and quantitative RT-PCR

Approximately 0.5 × 10^6^ RAW264.7 cells or BMDM were seeded in a 12-well plate and stimulated the next day with the indicated ligands for 24 h by addition to the media or permeabilization as describe in ref. ^5^ and ref. ^25^, respectively. Total RNA was subsequently isolated with TRI reagent (Sigma–Aldrich) as indicated by the manufacturer and cDNA was synthesized with iScript cDNA synthesis kit (Bio-Rad) and analyzed using a Bio-Rad CFX qRT-PCR. PCR array of immune gene groups was performed using PrimePCR Array plates using iTaq Universal SYBR Green Supermix (Bio-Rad). RNA-Seq was performed as previously described in ref. ^37^. Pathway analysis was done using the Ingenuity Pathway Analysis software (Qiagen). Primers used in this study are in Supplementary Table 1.

### Gel-filtration chromatography and mannosidase digestion

A 0.5 × 20 cm column was packaged with 6 ml of the BioGel P4 resin (Bio-Rad) and conditioned with a 25 mM NH_4_CH_3_COO (Sigma–Aldrich) buffer. Ten nanomoles of the total fOS were loaded into the column and 24 fractions (500 ul per fraction) were collected. Sample were dried in vacuum and later resuspended in water and desalted by ion exchange chromatography. Ten percent of each fraction was collected for FACE analysis, and the remaining 90% was divided in half and used for macrophage stimulation in biological duplicates. For the mannosidase digestion, 10 nmol of fOS or the synthetic ManGlcNAc disaccharide were treated with 40 U of α1-6 mannosidase (New England Biolabs) and 32 U of α1-2, 3 mannosidase (New England Biolabs). Samples were then mixed with 3 volumes of cold ethanol and centrifuged at 20,000 × *g* for 20 min at 4 °C. The supernatant was dried in vacuum and later resuspended in water and desalted by ion exchange chromatography. Ten percent of the digested product was collected for FACE analysis and the remaining 90% was divided in half and used for macrophage stimulation in biological duplicates.

### Structural analysis of free oligosaccharides

Free oligosaccharides in wild-type and *Trex1*^*−/−*^ cells were prepared as follows (also described in ref. ^11^). Briefly, 0.32 g of wild-type cells or 0.14 g of *Trex1*^*−/−*^ cells were resuspended with 800 ul of homogenization buffer (10 mM Hepes/NaOH buffer (pH 7.4) containing 5 mM dithiothreitol, 250 mM mannitol, 1 mM EDTA, 1 x complete protease inhibitor cocktail (EDTA-free; Roche Applied Science), and 1 mM of Pefabloc (Roche Applied Science)). After the cells were homogenized in a Potter-Elvehjem homogenizer, the supernatant of 100,000 × *g* centrifuged samples was obtained. The supernatant fractions were mixed with 3 volumes of cold ethanol, followed by centrifugation at 17,000 × *g* for 20 min at 4 °C. The supernatants were dried, and desalted using a PD-10 column (GE Healthcare). The free oligosaccaharide fraction thus obtained were subjected to fluorescence-labeling with 2-aminopyridine (PA). Detailed methods for PA-labeling, as well as removal of excess reagents were described previously^38^. Separation of PA-labeled free oligosaccaharides were carried out first by size-fractionation using a Shodex NH2P-50 3E column (3.0 × 250 mm; Shodex, Tokyo, Japan) as described previously^39^. Each PA-labeled glycans were further separated by a dual-gradient HPLC as described previously^40^. Structures of each PA-glycan isolated were confirmed by a co-elution of standard PA-glycans as prepared previously^11^.

### Mouse immunization study

C57BL/6 mice were transferred with mixed splenocytes (2 × 10^6^ for each) from OT1 and OT2 transgenic mice on day 1, and then immunized subcutaneously with 100 µg OVA together with/without 100 μg Manβ1-4GlcNAc or 20 μg LPS on day 0, 7, and 14. Seven days after the last immunization, serum of treated mice was collected for antibody detection with ELISA, and splenocytes were isolated for IFNγ ELISPOT assay. Serum anti-OVA IgG1 was measured using an ELISA kit per manufacturer’s protocol (Cayman #500830). Spleens from treated mice were processed into single cell suspensions and resuspended in RPMI 1640 medium supplemented with 10% fetal bovine serum, 2 mmol/l L-glutamine, 100 U/ml penicillin, and 100 μg/ml streptomycin. 5 × 10^5^ splenocytes were used for the IFNγ ELISPOT assay, 5 μg /ml of OVA (257-264), OVA (323-339), or SIY (SIYRYYGL) peptide were used to re-stimulate the antigen specific T cells. IFN-γ production was determined 48 h after of incubation with an IFN-γ ELISPOT assay kit according to the manufacturer’s protocol (BD Biosciences). The visualized spots were enumerated with the CTL-ImmunoSpot® S6 Analyzer (Cellular Technology Limited).

All mice were housed in pathogen-free barrier facility in UT Southwestern Medical Center. Both male and female 6–8 week old mice were used. All mice were on C57BL/6 background. Five mice per group were used in each repeat of the experiment. All mouse experiments were approved by ICUAC in UT Southwestern Medical Center.

### Glycan synthesis

See Supplementary Methods.

### Reporting summary

Further information on research design is available in the Nature Research Reporting Summary linked to this article.

## Supplementary information

Supplementary Information

Reporting Summary

Source Data

## Data Availability

All raw data, original gel images, processed RNA-seq data and pathway analysis are included in the Source Data file. Original RNA-seq data are deposited at NCBI gene expression omnibus (GEO, accession number GSE129677).
