# Supplementary material for: A bioactive mammalian disaccharide associated with autoimmunity activates STING-TBK1-dependent immune response
Source: Nat Commun. 2019 May 30;10:2377. doi: 10.1038/s41467-019-10319-5 (PMC6542856; doi:10.1038/s41467-019-10319-5)
Supplement: Supplementary file 1 — Supplementary Information [file 41467_2019_10319_MOESM1_ESM.pdf]

SUPPLEMENTARY INFORMATION

A bioactive mammalian disaccharide associated with autoimmunity activates STING-TBK1-dependent immune response

Nan Yan, et al

SUPPLEMENTARY FIGURES
SUPPLEMENTARY TABLE
SUPPLEMENTARY METHODS

SUPPLEMENTARY FIGURES

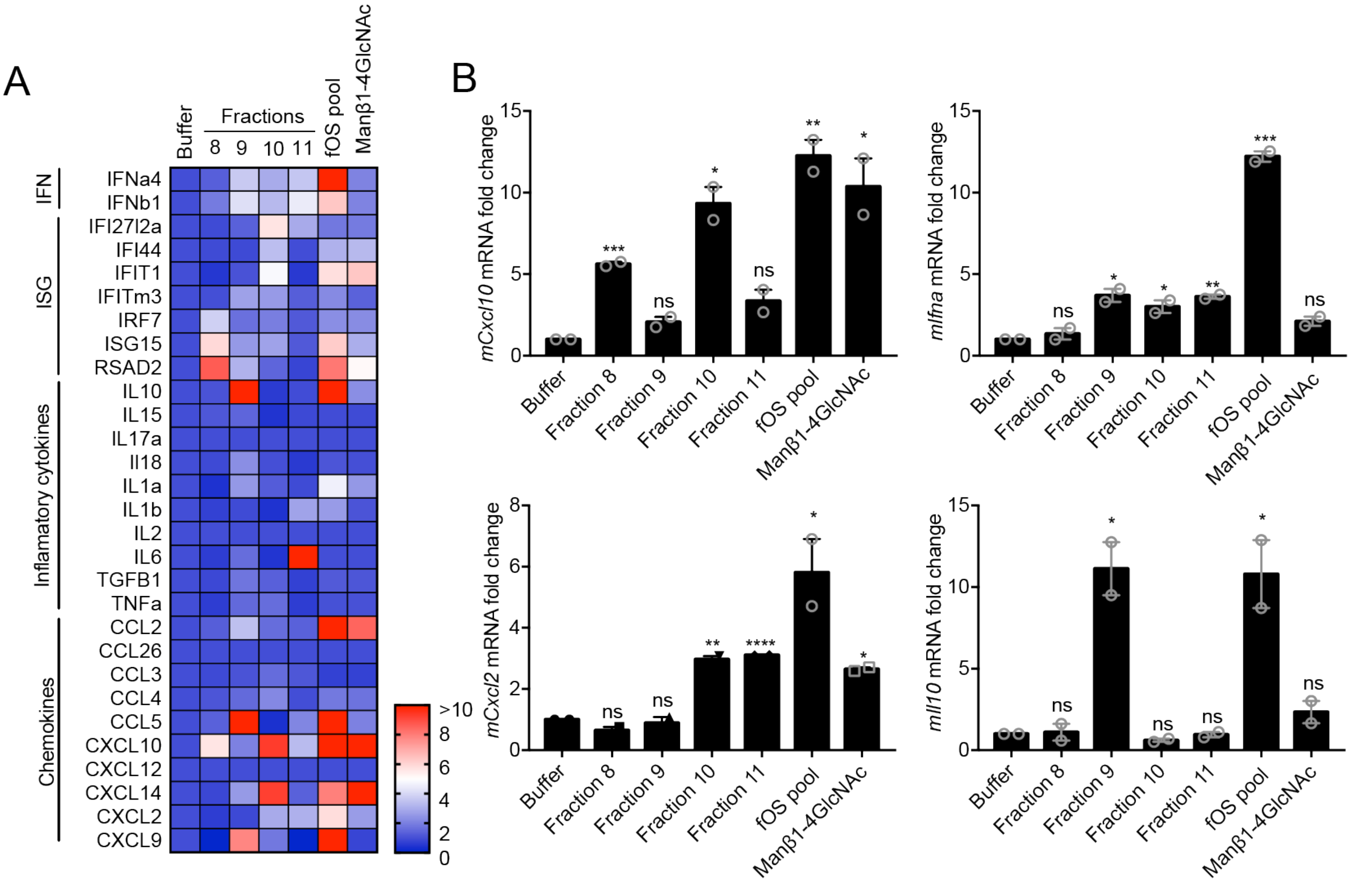

Supplementary Figure 1. fOS fractions stimulate distinct immune profiles.

Quantitative RT-PCR array analysis of immune gene expression induced by fOS fractions #8-11, fOS pool or Manβ1-4GlcNAc. Permeabilized BMDMs were treated with each fOS sample for 24 h. A heat map summarizing the data is shown in **A**. Representative immune genes are shown in **B**. Data are from a representative set of at least two independent experiments. Error bars indicate SEM. Unpaired t-test.

Glycan size	Abbreviations	Structure	Amount(pmol/g cells)	
			wild-type	*Trex1-/-*
Man1Gn1	M1A’	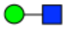	448	2391
Man2Gn1	M2A’	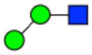	113	3299
Man3Gn1	M3D’	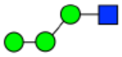	50	1001
Man4Gn1	M4D’	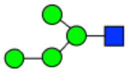	26	92
	M4E’	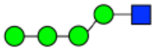	3	178

Supplementary Figure 2. Structural analysis and quantitation of WT and *Trex1*$^{-/-}$ fOS by two-dimensional HPLC.

HPLC trace plots are shown in **Figure 1B**. Top 5 fOS structures and quantitation are shown.

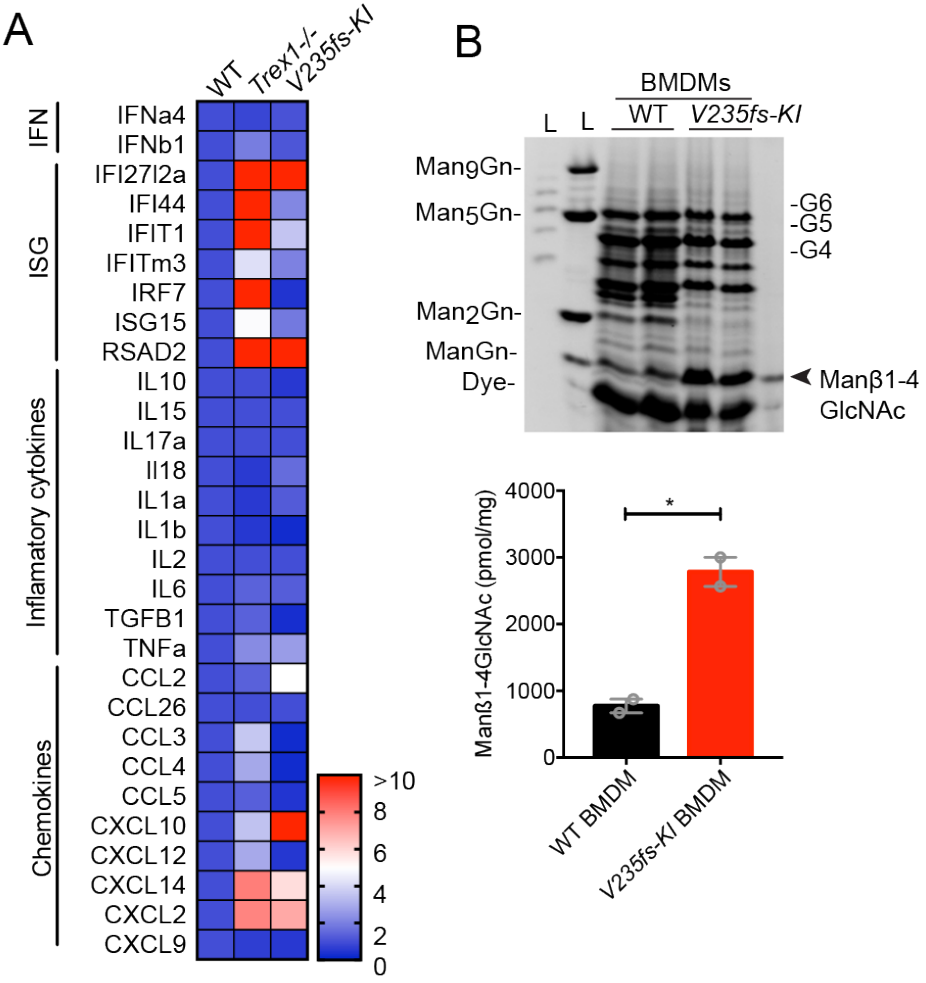

Supplementary Figure 3. The Manβ1-4GlcNAc disaccharide also accumulates in *TREX1-V235fs* mice.

(**A**) Quantitative RT-PCR array analysis of immune gene expression induced by fOS pool isolated from *Trex1*$^{-/-}$ or *TREX1-V235fs* BMDMs.

(**B**) FACE analysis of fOS isolated from WT and *TREX1-V235fs* BMDMs. Manβ1-4GlcNAc quantitation is shown in the lower panel. Data are from a representative set of at least two independent experiments. Error bars indicate SEM. Unpaired t-test.

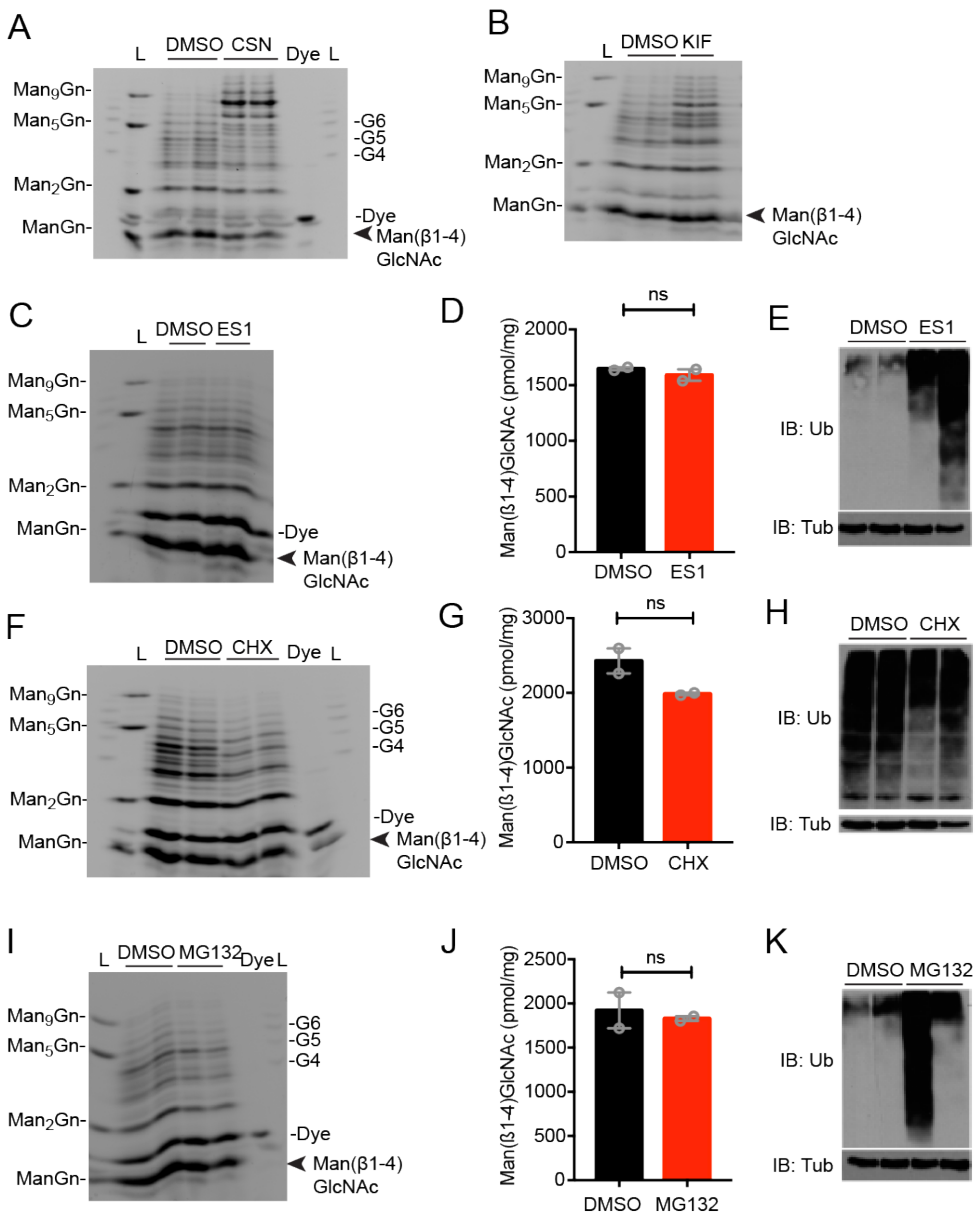

Supplementary Figure 4. Manβ1-4GlcNAc disaccharide biogenesis pathway.

(**A, B**) FACE analysis of *Trex1*$^{-/-}$ MEFs fOS treated with 100 µM CSN (**A**) or 100 µM KIF (**B**) for 24 h. (**C-K**) FACE analysis of *Trex1*$^{-/-}$ MEFs fOS treated with 10 µM ES1 (**C-E**), 10 µg/ml CHX (**F-H**), and 1 µM MG132 (**I-K**) for 24 h. Representative FACE gels are shown in panel **C, F, I.** Quantitation of Manβ1-4GlcNAc band are shown in panel **D, G, J.** Panel **E, H and K** are immunoblot analysis on total ubiquitin of *Trex1*$^{-/-}$ MEFs treated with ES1, CHX, and MG132. Treatment with ES1 and MG132 increases the total levels of ubiquitin while treatment with CHX decreases the total levels of ubiquitin. Error bars indicate SD. Unpaired t-test (**D, G, J**).

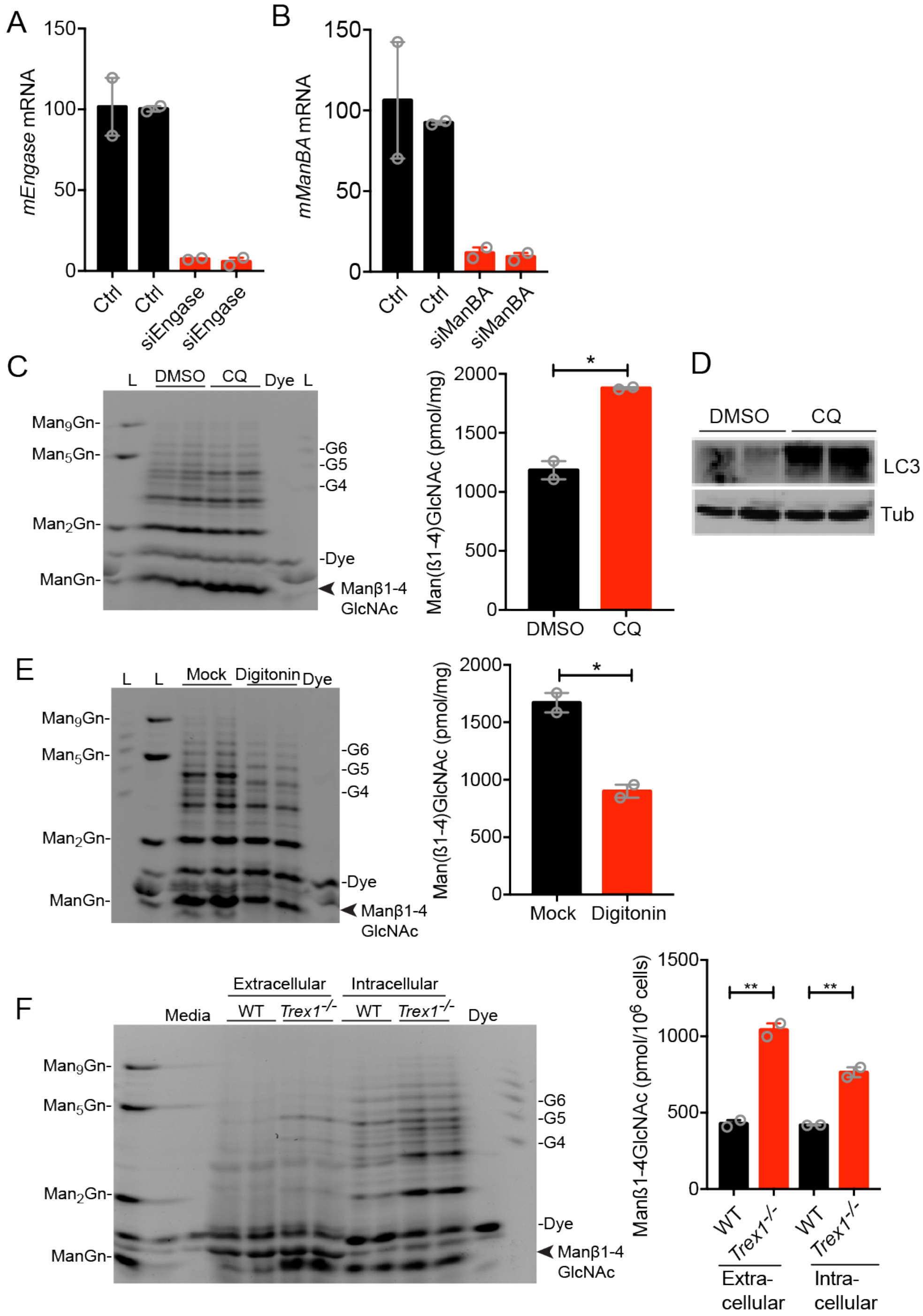

Supplementary Figure 5. The Manβ1-4GlcNAc disaccharide is produced through the cytoplasmic ENGase pathway and lysosomal catabolic pathway

(**A, B**) Quantitative RT-PCR analysis of ENGase (**A**) and MANBA (**B**) mRNA to confirm knockdown efficiency in *Trex1*$^{-/-}$ MEFs.

(**C**) FACE analysis of fOS pool isolated from *Trex1*$^{-/-}$ MEFs treated with 10 μM CQ for 24 h.

(**D**) Immunoblot analysis of LC3 and Tubulin (loading control) to confirm the efficiency of the CQ treatment in *Trex1*$^{-/-}$ MEFs.

(**E**) FACE analysis of fOS pool isolated from *Trex1*$^{-/-}$ MEFs treated with 10 μg/mL digitonin for 30 min prior methanol disruption.

(**F**) FACE analysis of fOS pool isolated from wild type (WT) or *Trex1*$^{-/-}$ MEFs cell lysates (intracellular) or culturing media (extracellular). Data are representative of at least three independent experiments. Error bars indicate SEM. Unpaired t-test.

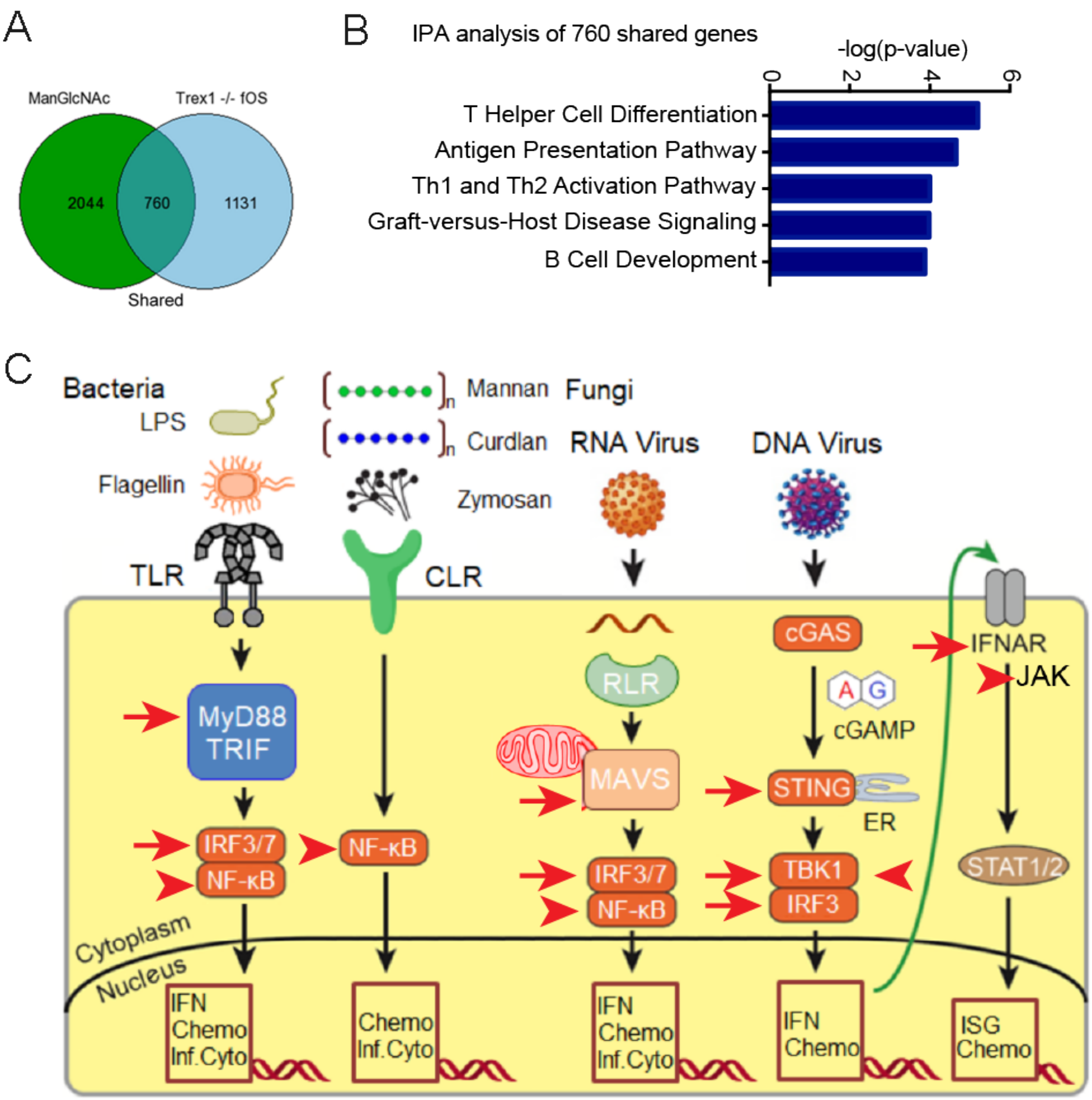

Supplementary Figure 6. Immune pathways activated by the Manβ1-4GlcNAc disaccharide.

(**A**) Venn diagram depicting the number of upregulated genes in the permeabilized RAW264.7 cells stimulated with the Manβ1-4GlcNAc (2044 genes), *Trex1*$^{-/-}$ fOS (1131 genes) and the number of upregulated genes that are shared (760) by both ligands.

(**B**) Ingenuity pathway analysis of enriched pathways using shared 760 genes that were induced by both Manβ1-4GlcNAc disaccharide and *Trex1*$^{-/-}$ fOS pool.

(**C**) A diagram showing innate immune signaling pathways and key components examined by gene knockout or inhibitor. Arrows, innate immune factors tested in knockout BMDM experiments in **Figure 5A**. Arrowheads, innate immune factors tested with inhibitors in **Figure 5B**.

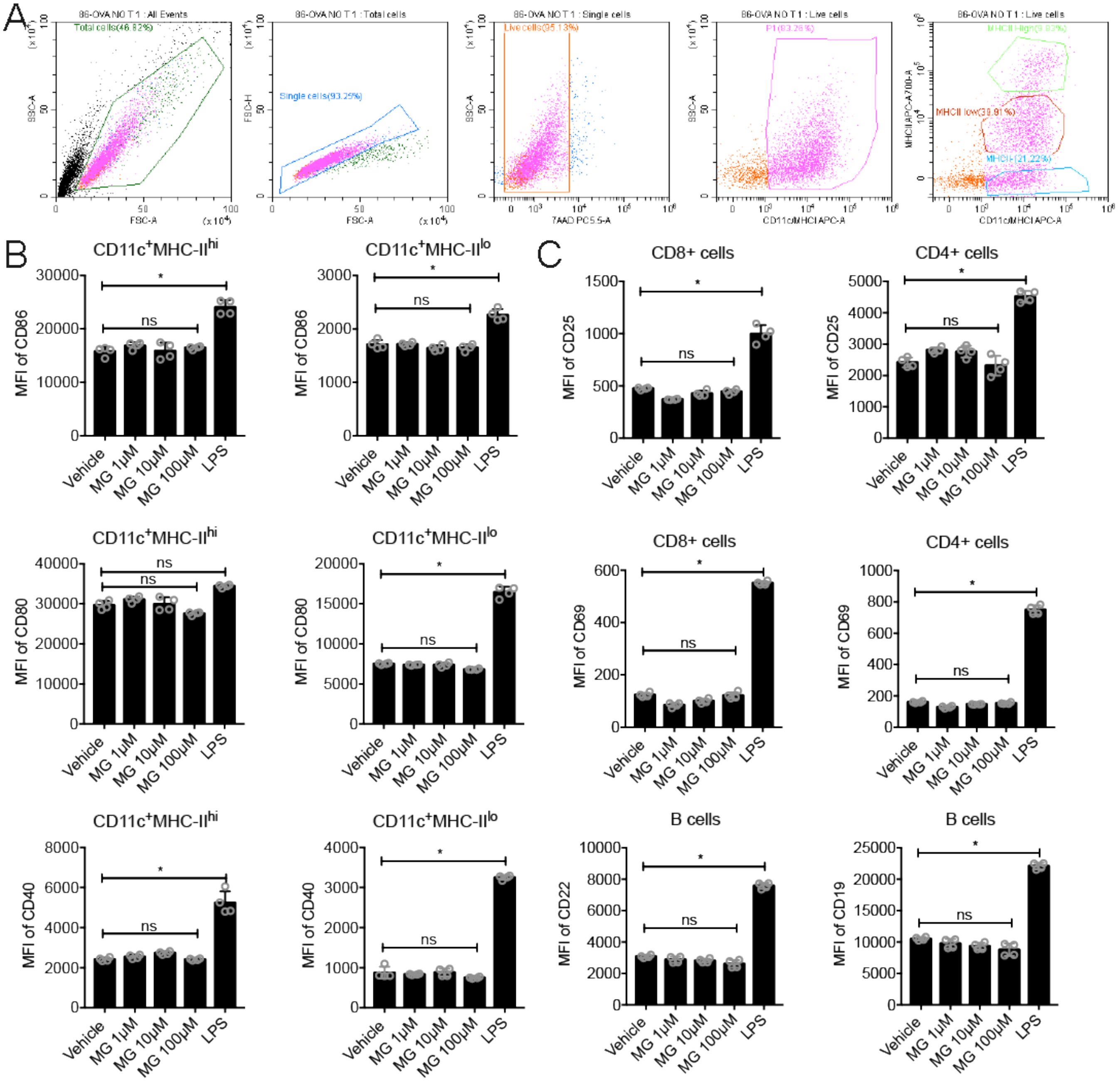

Supplementary Figure 7. Manβ1-4GlcNAc does not directly active BMDC, T and B cells.

(A) Gating strategy. **(B)** FACS analysis of CD86, CD80 and CD40 expression on CD11c+ MHC-II-high and -low DCs. **(C)** FACS analysis of CD25 and CD69 on CD4+ and CD8+ T cells and CD22 and CD19 on B cells. Data are representative of two independent experiments. Error bars indicate SD. Unpaired t-test.

Supplementary Table 1. DNA oligos used in this study.

All oligos were purchased from Sigma-Aldrich. qRT-PCR immune genes array plates (ISGs, inflammatory cytokines and chemokines) were purchased from Bio-Rad. Sequences can be requested directly from Bio-Rad. All oligos were validated by performing a Primer-Blast (https://www.ncbi.nlm.nih.gov/tools/primer-blast/index.cgi).

**Gene name**	**Forward and reverse oligo sequence used in qRT-PCR analyses**
Mouse *Gapdh*	TTCACCACCATGGAGAAGGC<br>GGCATCGACTGTGGTCATGA
Mouse *Ifit1*	GAACCCATTGGGGATGCACAACCT<br>CTTGTCCAGGTAGATCTGGGCTTCT
Mouse *Cxcl10*	GGGATCCCTCTCGCAAGGACGGTCC<br>ACGCTTTCATTAAATTCTTGATGGT
Mouse *Cxcl2*	CGGTCAAAAAGTTTGCCTTG<br>TCCAGGTCAGTTAGCCTTGC
Mouse *Il10*	AGAAAAGAGAGCTCCATCATGC<br>TTATTGTCTTCCCGGCTGTACT
Mouse *ENGase*	ACCTCTGAGCCCTGAATGAA<br>TTCAATGTAGCCCTGGAACC
Mouse *ManBA*	AAGCAGCCAAGGTTTATTTC<br>ATCCAATTTGAGCCTTTCAG

SUPPLEMENTARY METHODS

1. Chemical synthesis

1-1. General information

1-2. **Supplementary Figure 8.** Synthesis of disaccharide **4α** and **4β**

1-3. **Supplementary Figure 9.** Synthesis of trisaccharide **10**

1-4. Experimental Procedure

1-5. NMR Spectra of Compounds **3α**, **3β**, **4α**, **4β**, **7**, **9**, and **10**

1. Chemical synthesis

1-1. General information

All reactions sensitive to moisture were carried out under argon atmosphere with anhydrous solvents under anhydrous conditions, unless otherwise noted. These solvents and other reagents were purchased from Kanto Chemical Co., Inc (Tokyo, Japan), Tokyo Chemical Industry (Tokyo, Japan) and Wako Pure Chemical Industries Ltd. (Tokyo, Japan) and used without further purification, unless otherwise noted. Analytical thin layer chromatography was developed on TLC Silica gel 60 F_{254} plate (Merck, Darmstadt, Germany). Silica gel column chromatography was performed on Silica gel 60 N (40–100 mesh or 100–210 mesh, Kanto Chemical Co., Inc, Tokyo, Japan) or Hi-Flash column (YAMAZEN, Osaka, Japan). MALDI-TOF MS was recorded in the high-resolution mode with positive ion mode on an AXIMA-Performance (Shimadzu, Kyoto, Japan). NMR spectra were recorded with JNM-ECA600 (JEOL, Tokyo, Japan; ^{1}H: 600 MHz, ^{13}C: 150 MHz). Chemical shifts are given in ppm and referenced to internal TMS (δH 0.00 in $CDCl_3$), $CDCl_3$ (δC 77.16 in $CDCl_3$) or HOD (δH 4.65 in D_2O). The resonances were assigned according to diagram below.

Manβ1-4GlcNAc
II I

Manα1-3Manβ1-4GlcNAc
III II I

1-2. Supplementary Figure 8. Synthesis of disaccharide **4α** and **4β**

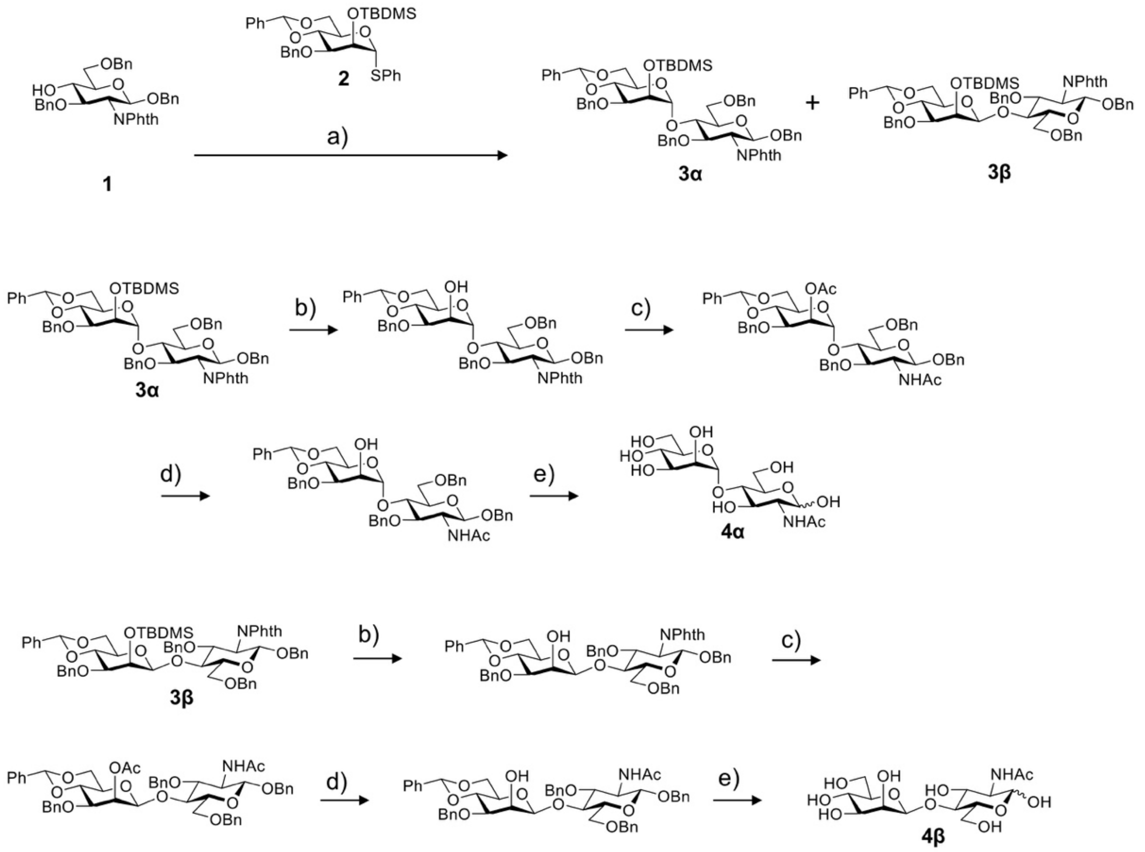

Supplementary Figure 8. Reagents and conditions; a) NIS/TfOH, MS4A, CH_2Cl_2, b) 1M TBAF, THF, c) i) ethylenediamine, *n*-BuOH, ii) Ac_2O, pyridine; 40 °C, d) 1M NaOMe, MeOH, e) $Pd(OH)_2/C$, THF/H_2O.

1-3. Supplementary Figure 9. Synthesis of trisaccharide **10**

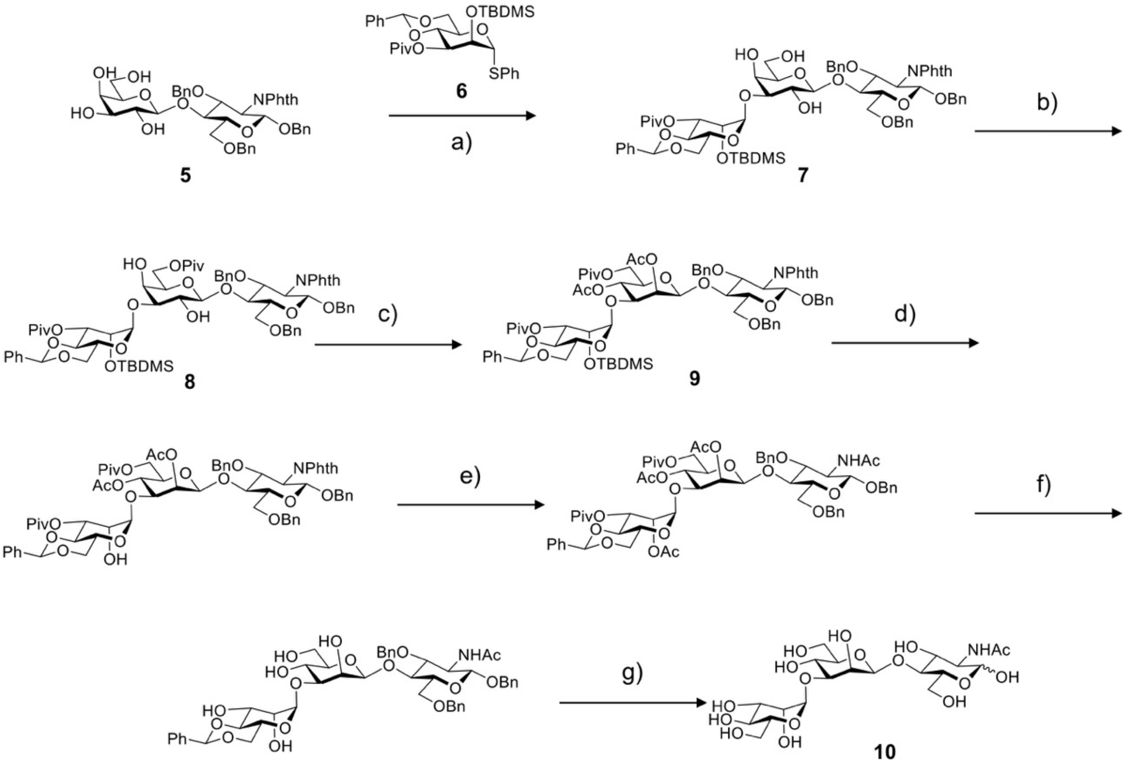

Supplementary Figure 8. Reagents and conditions; a) NIS/TfOH, MS4A, CH_2Cl_2, b) PivCl, pyridine, c) i) Tf_2O, pyridine, ii) CsOAc, 18-crown-6, d) 1M TBAF, THF, e) i) ethylenediamine, *n*-BuOH, ii) Ac_2O, pyridine, f) 1M NaOMe, MeOH, g) $Pd(OH)_2/C$, THF/H_2O.

1-4. Experimental Procedure

Benzyl 3-*O*-benzyl-4,6-*O*-benzylidene-2-*O*-*tert*-butyldimethylsilyl-α-D-mannopyranosyl-(1→4)-3,6-di-*O*-benzyl-2-deoxy-2-phthalimido-β-D-glucopyranoside (**3α**) and Benzyl 3-*O*-benzyl-4,6-*O*-benzylidene-2-*O*-*tert*-butyldimethylsilyl-β-D-mannopyranosyl-(1→4)-3,6-di-*O*-benzyl-2-deoxy-2-phthalimido-β-D-glucopyranoside (**3β**)

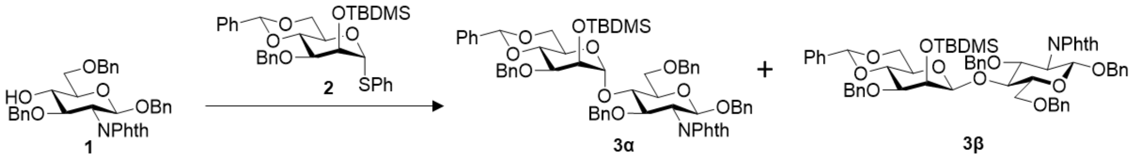

A mixture of glycosyl acceptor **1** (100 mg, 0.18 mmol), glycosyl donor **2** (154 mg, 0.27 mmol), NIS (99.4 mg, 0.44 mmol) and MS4 Å (750 mg) was dissolved in 7 mL of dried CH_2Cl_2 under argon. TfOH (8 µL, 90 µmol) was added to the mixture at -78 °C. The reaction mixture was stirred at -50 °C for 3 hours, before the reaction was quenched with Et_3N (25 µL, 180 µmol) at -50 °C. The reaction mixture was diluted with EtOAc, and filtered through celite. The filtrate was washed with aq. $Na_2S_2O_3$, saturated aq. $NaHCO_3$, and brine. The organic layer was dried over anhydrous $MgSO_4$, filtered, and concentrated *in vacuo*. The resulting residue was purified by gel filtration chromatography with toluene and by flash silica gel column chromatography with toluene-EtOAc (12/1, *v/v*) to give **3α** (74.1 mg, 72 µmol, 40%) as a colorless syrup, **3β** (79.5 mg, 77 µmol, 43%) as a colorless syrup; **3α**; R_f = 0. 55 (Toluene/EtOAc = 12/1, *v/v*); ^{1}H NMR (600 MHz, $CDCl_3$): δ 7.63-6.76 (m, 29H, aromatic *H*), 5.64 (s, 1H, PhC*H*), 5.07 (m, 2H, H-1^{I}, H-1^{II}), 4.77 (m, 2H, PhCH_2), 4.71 (d, 1H, *J* =12.6 Hz, PhCH_2), 4.65 (m, 2H, PhCH_2), 4.60 (d, 1H, *J* = 12.0 Hz, PhCH_2), 4.47 (d, 1H, *J* = 12.6 Hz, PhCH_2), 4.22 (m, 6H, H-2^{I}, H-3^{I}, H-2^{II}, H-6^{II}, H-6^{II}, PhCH_2), 3.92 (m, 2H, H-4^{I}, H-5^{II}), 3.83 (m, 4H, H-6^{I}, H-6^{I}, H-3^{II}, H-4^{II}), 3.59 (dt, 1H, *J* = 3.0 Hz, *J* = 6.6 Hz, *J* = 10.2 Hz, H-5^{I}), 0.922 (s, 1H, $Si(CH_3)_2C(CH_3)_3$), 0.090, 0.082 ppm (s, 6H, $Si(CH_3)_2C(CH_3)_3$). ;^{13}C NMR (150 MHz, $CDCl_3$): δ 138.65-123.26 (aromatic *C*), 103.95 (C-1^{II}), 101.64 (Ph*C*H), 97.18 (C-1^{I}), 80.22, 79.38, 78.82, 75.43, 74.89, 74.42, 73.60, 72.98, 71.85, 70.76, 69.01, 65.45, 55.61, 25.86 ($Si(CH_3)_2C(CH_3)_3$), 18.60 ($Si(CH_3)_2C(CH_3)_3$), -4.279, -5.083 ppm (2C, $Si(CH_3)_2C(CH_3)_3$).; MALDI-TOF MS: *m/z* calcd for $C_{61}H_{67}NNaO_{12}Si^+$: 1056.433 $[M+Na]^+$; found: 1056.678; **3β**; R_f = 0. 20 (Toluene/EtOAc = 12/1, *v/v*); ^{1}H NMR (600 MHz, $CDCl_3$): δ 7.49-6.75 (m, 29H, aromatic *H)*, 5.50 (s, 1H, PhC*H*), 5.14 (d, 1H, $J_{1,2}$ = 8.4 Hz, H-1^{I}), 4.88 (d, 1H, *J* = 12.0 Hz, PhCH_2), 4.79 (m, 2H, PhCH_2), 4.70 (d, 1H, *J* = 12.0 Hz, PhCH_2), 4.66 (d, 1H, *J* =12.0 Hz, PhCH_2), 4.47 (m, 3H, PhCH_2, H-1^{II}), 4.34 (d, 1H, *J* = 12.6 Hz, PhCH_2), 4.24 (m, 2H, H-2^{I}, H-3^{I}), 4.14 (dd, 1H,

$J_{5,6}$ = 4.8 Hz, $J_{6,6'}$ = 10.2 Hz, H-6^{II}), 4.01 (dd, 1H, $J_{3,4}$ = 8.4 Hz, $J_{4,5}$ = 10.2 Hz, H-4^{I}), 3.97 (t, 1H, $J_{3,4}$ = $J_{4,5}$ = 9.0 Hz, H-4^{II}), 3.90 (d, 1H, $J_{1,2}$ = 2.4 Hz, H-2^{II}), 3.77 (dd, 1H, $J_{5,6}$ = 2.4 Hz, H-6^{I}), 3.72 (dd, 1H, $J_{5,6'}$ = 3.6 Hz, $J_{6,6'}$ = 10.2 Hz, H-6'^{I}), 3.56 (td, 1H, $J_{4,5}$ = 10.2 Hz, $J_{5,6}$ = 2.4 Hz, $J_{5,6'}$ = 3.6 Hz, H-5^{I}), 3.51 (t, 1H, $J_{5,6}$ =$J_{6,6'}$ = 10.2 Hz, H-6'^{II}), 3.32 (dd, 1H, $J_{2,3}$ = 2.4 Hz, $J_{3,4}$ = 9.6 Hz, H-3^{II}), 3.14 (td, 1H, $J_{5,6}$ = 4.8 Hz, $J_{6.6'}$ = 10.2 Hz, H-5^{II}), 0.934 (s, 1H, Si(CH$_3$)$_2$C(C*H$_3$)*$_3$), 0.129, 0.075 ppm (s, 6H, Si(C*H*$_3$)$_2$C(CH$_3$)$_3$); ^{13}C NMR (150 MHz, CDCl$_3$): δ 139.07-123.31 (aromatic *C*), 102.09 (C-1^{II}), 101.48 (Ph*C*H), 97.62 (C-1^{I}), 80.45, 79.07, 78.12, 77.44, 75.04, 74.97, 73.80, 72.88, 72.14, 70.90, 68.78, 68.75, 67.30, 55.87, 26.13 (Si(CH$_3$)$_2$C(*C*H$_3$)$_3$), 18.60 (Si(CH$_3$)$_2$*C*(CH$_3$)$_3$), -3.656, -4.346 ppm (2C, Si(*C*H$_3$)$_2$C(CH$_3$)$_3$) ; MALDI-TOF MS: *m/z* calcd for: $C_{61}H_{67}NNaO_{12}Si^+$: 1056.433 [M+Na]$^+$; found: 1056.497.

α-D-mannopyranosyl-(1-4)-2-acetamido-2-deoxy-D-glucopyranose (**4α**)

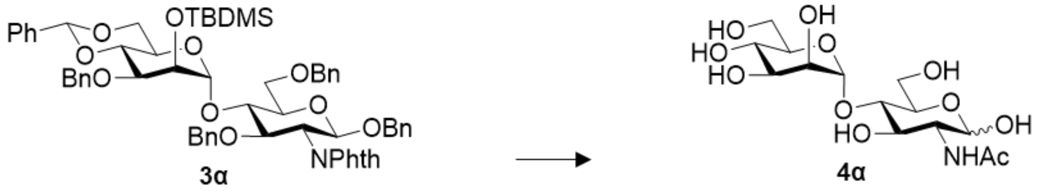

To a solution of compound **3α** (74 mg, 72 μmol) in THF (1.4 mL) was added 1M TBAF in THF (220 μL, 216 mmol) at 0 °C. The reaction mixture was stirred at 40 °C for 8 h. The reaction mixture was concentrated *in vacuo*. The residue was dissolved in *n*-BuOH (1.6 mL) and then ethylenediamine (480 μL, 7.2 mmol) was added. The reaction mixture was stirred at 90 °C for 1 day. The reaction mixture was concentrated *in vacuo*. The residue was dissolved in pyridine (2 mL), and Ac_2O (300 μL) was added at room temperature. The reaction mixture was stirred at 40 °C for 1 day, and then quenched with MeOH. The solution was concentrated *in vacuo*. The residue was diluted with $CHCl_3$, washed with 1M HCl, brine, saturated aq. $NaHCO_3$, and brine, successively. The organic layer was dried with $MgSO_4$, filtered, and evaporated *in vacuo*. The residue was dissolved in THF (1 mL) and MeOH (0.5 mL). The solution was added 1M NaOMe in MeOH (270 μL) at 0 °C. The reaction mixture was stirred 40 °C for 3 hours, and neutralized with Amberlyst 15DRY, filtered, and concentrated *in vacuo*. The resulting residue was purified by flash silica gel column chromatography with chloroform/MeOH (20/1, *v/v*) to afford intermediate (37 mg, 62% in 4 steps). The compound was dissolved in THF (3 mL) and H_2O (3 mL), stirred in the presence of $Pd(OH)_2/C$ (26 mg) at 40 °C under H_2 atmosphere for 1 day. The reaction mixture was filtered through celite. The filtrate was lyophilized. The residue was purified by ISOLUT C18 (H_2O) to afford compound **4α** (11 mg, 29 μmol, 40% in 5 steps); R_f = 0.53 (CH_3CN/H_2O=2/1, *v/v*); ^{1}H NMR (600 MHz, D_2O): δ 5.17 (d, 1H, J = 1.2 Hz; H-1^{II}), 5.06 (d, 1H, J = 2.4 Hz; H-$1\alpha^{I}$), 4.58 (m, 1H, H-$1\beta^{I}$), 3.92 (ddd, 1H, J = 1.8 Hz, J = 3.0 Hz , J = 13.8 Hz), 3.83-3.41 (m, 11H), 1.92 (s, 3H,COCH_3), 1.92 ppm (s, 3H, COCH_3).; MALDI-TOF-MS: *m*/*z* calcd for $C_{14}H_{25}NNaO_{11}^+$: 406.132; found: 406.083.

β-D-mannopyranosyl-(1-4)-2-acetamido-2-deoxy-D-glucopyranose (**4β**)

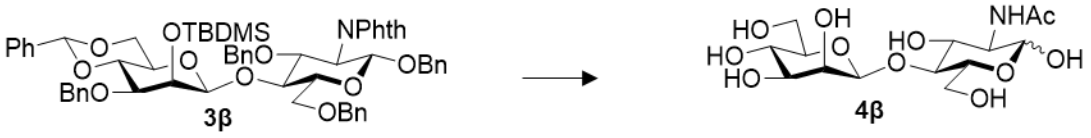

To a solution of compound **3β** (80 mg, 77 μmol) in THF (1.4 mL) was added 1M TBAF in THF (230 μL, 237 mmol) at 0 °C. The reaction mixture was stirred at 40 °C for 1 day. The reaction mixture was concentrated *in vacuo*. The residue was dissolved in *n*-BuOH (1.7 mL) and then ethylenediamine (500 μL, 7.7 mmol) was added. The reaction mixture was stirred at 90 °C for 1 day. The reaction mixture was concentrated *in vacuo*. The residue was dissolved in pyridine (1 mL), and Ac_2O (300 μL) was added at room temperature. The reaction mixture was stirred at 40 °C for 1 day, and then quenched with MeOH. The solution was concentrated *in vacuo*. The residue was diluted with chloroform, washed with 1M HCl, brine, saturated aq. $NaHCO_3$, and brine, successively. The organic layer was dried with $MgSO_4$, filtered, and evaporated *in vacuo*. The residue was dissolved in THF (1 mL) and MeOH (0.5 mL). The solution was added 1M NaOMe in MeOH (370 μL) at 0 °C. The reaction mixture was stirred 40 °C for 12 hours, and neutralized with Amberlyst 15DRY, filtered, and concentrated *in vacuo*. The resulting residue was purified by flash silica gel column chromatography with chloroform/MeOH (20/1, *v/v*) to afford intermediate (70 mg, quant. in 4 steps). The compound was dissolved in THF (5 mL) and H_2O (5 mL), stirred in the presence of $Pd(OH)_2/C$ (50 mg) at 40 °C under H_2 atmosphere for 1 day. The reaction mixture was filtered through celite. The filtrate was lyophilized. The residue was purified by ISOLUT C18 (H_2O) to afford compound **4β** (22 mg, 57 μmol, 67%) ;R_f = 0.53 (CH_3CN/H_2O=2/1, *v/v*); ^{1}H NMR (600 MHz, D_2O): δ 5.28 (d, J = 3.0 Hz, H-1α^{I}), 4.83 (s, 1H, H-1^{II}), 4.79 (d, J = 7.8 Hz, H-1β^{I}, 4.14-3.47 (m, 12H), 2.11 ppm (s, 3H, $COCH_3$).; MALDI-TOF MS: *m/z* calcd for $C_{14}H_{25}NNaO_{11}^+$: 406.132; found: 406.117.

Benzyl14,6-*O*-benzylidene-3-*O*-pivaloyl-2-*O*-*tert*-butyldimethylsilyl-α-D-mannopyranosyl-(1-3)-β-D-galactopyranosyl-(1-4)-3,6-di-*O*-benzyl-2-deoxy-2-phtalimide-β-D-glucopyranoside (**7**)

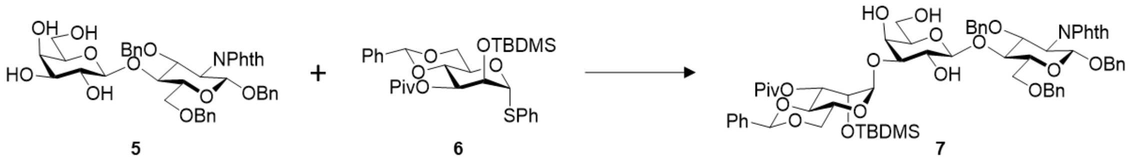

A mixture of glycosyl acceptor **5** (100 mg, 135 μmol), glycosyl donor **6** (165 mg, 297 μmol), NIS (46 mg, 203 μmol) and MS4 Å (800 mg) was dissolved in 16 mL of dried CH_2Cl_2 (16 mL) under argon. TfOH (13 μL, 149 μmol) was added to the mixture at -78 °C. The reaction mixture was stirred at -78 °C for 12 hours, before the reaction was quenched with Et_3N (206 μL, 1.49 mmol) at -78 °C. The reaction mixture was diluted with EtOAc, and filtered through celite. The filtrate was washed with aq. $Na_2S_2O_3$, saturated aq. $NaHCO_3$, and brine. The organic layer was dried over anhydrous $MgSO_4$, filtered, and concentrated *in vacuo*. The resulting residue was purified by gel filtration chromatography with toluene and by flash silica gel column chromatography with hexane-EtOAc (hexane/EtOAc = 1/1, *v/v*) to give **7** (91 mg, 87 μmol, 64%) as a white powder; R_f = 0.33 (Hexane/EtOAc = 1/1, *v/v*); ^{1}H NMR (600 MHz, $CDCl_3$): δ 7.65-6.84 (m, 24H, aromatic *H*), 5.60 (s, 1H, PhC*H*), 5.35 (dd, 1H, $J_{2,3}$ = 3.0 Hz, $J_{3,4}$ = 10.2 Hz, H-3^{III}), 5.13 (d, 1H, $J_{1,2}$ = 8.4 Hz, H-1^{I}), 4.83 (m, 3H, H-1^{III}, PhCH_2), 4.73 (d, 1H, *J* = 12.0 Hz, PhCH_2), 4.59 (d, 1H, *J* = 12.0 Hz, PhCH_2), 4.49 (m, 3H, H-1^{II}, PhCH_2), 4.37 (dd, 1H, *J* = 8.4 Hz, *J* = 10.8 Hz, H-3^{I}), 4.19 (m, 6H, H-2^{I}, H-4^{I}, H-2^{III}, H-4^{III}, H-5^{III}, H-6^{III}), 4.02 (dd, 1H, *J* = 3.6 Hz, *J* = 11.4 Hz, H-6^{I}), 3.82 (m, 4H, H-6^{I}, H-2^{II}, H-4^{II}, H-6^{III}), 3.66 (m, 3H, H-5^{I}, H-6^{II}, H-6^{II}), 3.49 (dd, 1H, *J* = 3.0 Hz, *J* = 9.6 Hz, H-3^{II}), 3.22 (m, 2H, H-5^{II},-O*H*), 2.71 (O*H*), 1.80 (O*H*), 1.20 (s, 9H, COC(CH_3)$_3$), 0.943 (Si(CH$_3$)$_2$C(CH_3)$_3$), 0.070, 0.068 ppm (s, 6H, Si(CH_3)$_2$C(CH$_3$)$_3$). ; ^{13}C NMR (150 MHz, $CDCl_3$): δ 178.26 (*C*OC(CH$_3$)$_3$), 138.52-126.00 (aromatic *C*), 103.15 (C-1^{II}), 101.28 (Ph*C*H), 98.56 (C-1^{III}), 97.59 (C-1^{I}), 78.53, 78.36, 77.67, 76.29, 74.84, 74.72 (Ph*C*H$_2$), 74.19, 73.56 (Ph*C*H$_2$), 71.01, 70.89 (Ph*C*H$_2$), 70.64, 70.39, 68.83 (C-6^{III}), 68.33 (C-6^{I}), 66.68, 64.87, 62.74 (C-6^{II}), 55.97 (C-2^{I}), 39.10 (CO*C*(CH$_3$)$_3$), 27.36 (COC(*C*H$_3$)$_3$), 25.80 (Si(CH$_3$)$_2$C(*C*H$_3$)$_3$), 18.04 (Si(CH$_3$)$_2$*C*(CH$_3$)$_3$), -4.48, -4.72 ppm (Si(*C*H$_3$)$_2$C(CH$_3$)$_3$); MALDI-TOF MS: *m*/*z* calcd for $C_{65}H_{79}NNaO_{18}Si^+$: 1212.496[M+Na]$^+$, found: 1212.413.

Benzyl 3-*O*-benzyl-4,6-*O*-benzylidene-2-*O*-*tert*-butyldimethylsilyl-α-D-mannopyranosyl-(1-3)-2,4-di-*O*-acetyl-6-*O*-pivaloyl-β-D-mannopyranosyl-(1-4)-3,6-di-*O*-benzyl-2-deoxy-2-phthalimido-β-D-glucopyranoside (**9**)

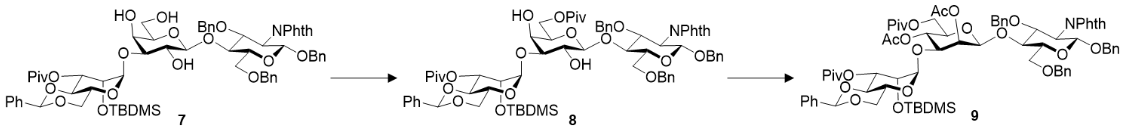

To a solution of compound **7** (100 mg, 84 μmol) in pyridine (340 μL) was added pivaloyl chloride (52 μL, 420 μmol) at 0 °C. The reaction mixture was stirred at room temperature for 2 hours, and before the reaction was quenched with methanol, azetropic-dried 3 times, diluted with EtOAc and washed with 1M HCl, brine, saturated aq. $NaHCO_3$, and brine, successively. The organic layer was dried with $MgSO_4$, filtered, and evaporated *in vacuo*. The resulting residue was purified by flash silica gel column chromatography with toluene-EtOAc (Toluene/EtOAc = 4/1, *v/v*) to give **8** (104 mg, 92 μmol, quant.). A solution of compound **8** (93 mg, 73 μmol) in CH_2Cl_2 (1.3 mL) was added trifluoromethanesulfonic anhydrade (123 μL, 730 μmol) and dry pyridine (176 μL, 2.19 mmol) at 0 °C. The reaction mixture was stirred at room temperture for 30 min, and before the reaction was quenched with $NaHCO_3$ aq. and diluted with EtOAc and washed with brine. The organic layer was dried over anhydrous $MgSO_4$, filtered, and concentrated *in vacuo*. The resulting residue was purified by gel filtration chromatography with toluene and by flash silica gel column chromatography with toluene-EtOAc (Toluene/EtOAc = 8/1, *v/v*). The product was azetropic-dried 3 times with dried toluene. A solution of in dry toluene (2.8 mL) was added CsOAc (140 mg, 730 μmol) and 18-crown-6 (193 mg, 730 μmol). The reaction mixture was kept in a water bath under ultrasonication for overnight. The reaction mixture was diluted with EtOAc, and then the organic layer was washed with Brine, sat. aq. $NaHCO_3$, Brine, dried over anhydrous $MgSO_4$, filtered, concentrated *in vacuo*. The resulting residue was purified by flash silica gel column chromatography with toluene-EtOAc (4:1 *v/v*) to give **9** (48 mg, 34 μmol, 47% in 3 steps); R_f = 0. 50 (Toluene/EtOAc = 4/1, *v/v*); ^{1}H-NMR (600 MHz, $CDCl_3$) δ 7.79-6.65 (m, 24H), 5.55 (s, 1H, PhC*H*), 5.39 (d, 1H, $J_{1,2}$ = 3.0 Hz, H-2^{II}), 5.15 (m, 2H, H-4^{II}, H-3^{III}), 5.05 (d, 1H, $J_{1,2}$ = 7.8 Hz, H-1^{I}) 4.83 (d, 1H, *J* = 12.6 Hz, PhCH_2), 4.77 (m, 2H, PhCH_2), 4.70 (d, 1H, $J_{1,2}$ = 1.8 Hz, H-1^{III}), 4.67 (s, 1H, H-1^{II}), 4.47 (m, 2H, PhCH_2), 4.37 (d, 1H, *J* = 12.6Hz, PhCH_2), 4.29 (dd, 1H, $J_{6,6'}$ = 4.2 Hz, $J_{5,6}$ = 10.2 Hz, H-6^{III}), 4.08 (m, 7H, H-2^{I}, H-3^{I}, H-4^{I}, H-6^{II}, H-6^{II}, H-2^{III}, H-4^{III}), 3.87 (m, 2H, H-6^{I}, H-5^{III}), 3.78 (m, 2H, H-6^{I}, H-6^{III}), 3.53 (m, 2H, H-5^{I}, H-3^{II}), 3.33 (qd, 1H, *J* = 1.8 Hz, *J* = 4.8 Hz, *J* = 7.2 Hz, H-5^{II}), 2.18, 2.08 (s, 3H, COCH_3), 1.18, 1.15 (s, 9H, COC(CH_3)$_3$), 0.964 (s, 9H, Si(CH$_3$)$_2$C(CH_3)$_3$), 0.080, 0.073 ppm (s, 3H, Si(CH_3)$_2$C(CH$_3$)$_3$).; ^{13}C NMR (150 MHz, $CDCl_3$): δ 178.23, 177.78, 170.02, 169.16, 167.65, 167.58, 138.65-123.18 (aromatic *C*), 103.67 (C-1^{III}), 101.52 (PhCH), 98.83 (C-1^{II}), 97.45 (C-1^{I}), 79.44, 77.68, 76.49, 76.18, 74.66, 74.49, 73.62, 73.46, 72.21, 70.91, 70.54, 68.83 (C-6^{III}), 68.42 (C-6^{I}), 67.60, 65.53, 62.00 (C-6^{II}), 55.78 (C-2^{I}), 39.05, 38.92 (2C, CO*C*(CH$_3$)$_3$), 27.39, 27.13 (2C,

COC(CH_3)$_3$), 25.81 (Si(CH_3)$_2$C(CH_3)$_3$), 20.99, 18.07 (2C, COCH_3), -4.470, 4.643 ppm (2C, s, 3H, Si(CH_3)$_2$C(CH_3)$_3$). ; MALDI-TOF MS: *m*/*z* calcd for $C_{74}H_{91}NNaO_{21}Si^+$: 1380.575; found: 1380.749.

α-D-mannopyranosyl-(1-3)-β-D-mannopyranosyl-(1-4)-2-acetamido-2-deoxy-D-glucopyranose (**10**)

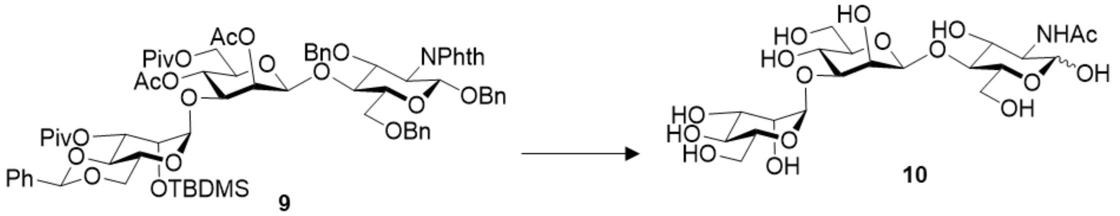

To a solution of compound **9** (48 mg, 34 µmol) in THF (700 µL) was added 1M TBAF in THF (102 µL, 102 µmol) at 0 °C. The reaction mixture was stirred at 40 °C for 1 day. The reaction mixture was concentrated *in vacuo*. The residue was dissolved in *n*-BuOH (760 µL) and then ethylenediamine (227 µL, 3.4 mmol) was added. The reaction mixture was stirred at 90 °C for 4 days. The reaction mixture was concentrated *in vacuo*. The residue was dissolved in pyridine (1 mL), and Ac_2O (150 µL) was added at room temperature. The reaction mixture was stirred at 40 °C for 4 days, and then quenched with MeOH. The solution was concentrated *in vacuo*. The residue was diluted with chloroform, washed with 1M HCl, brine, saturated aq. $NaHCO_3$, and brine, successively. The organic layer was dried with $MgSO_4$, filtered, and evaporated *in vacuo*. The residue is dissolved in THF (0.5 mL) and MeOH (0.3 mL). The solution was added 1M NaOMe in MeOH (130 µL) at 0 °C. The reaction mixture was stirred 40 °C for 2 days., and neutralized with Amberlyst 15DRY, filtered, and concentrated *in vacuo*. The resulting residue was purified by flash silica gel column chromatography with chloroform/MeOH (20/1, *v/v*) to afford intermediate (31 mg, 62% in quant. in 4 steps). The compound was dissolved in THF (2 mL) and H_2O (2 mL), stirred in the presence of $Pd(OH)_2/C$ (20 mg) at 40 °C under H_2 atmosphere for 4 days. The reaction mixture was filtered through celite. The filtrate was lyophilized. The residue was purified by ISOLUT C18 (H_2O) to afford compound **10** (14 mg, 26 µmol, 76% in 5 steps); R_f = 0.53 (CH_3CN/H_2O=2/1, *v/v*); ^{1}H NMR (600 MHz, D_2O): δ 5.27 (d, *J* = 3.0 Hz, H-1α^I), 5.18 (s, 1H, H-1), 4.85 (s, 1H, H-1), 4.78 (d, 1H, *J* = 7.8 Hz, H-1β^I), 4.29 (dd, 1H, *J* = 3.0 Hz, *J* = 6.6 Hz), 4.14 (bt, 1H, *J* = 1.8 Hz), 4.01-3.35 (m, 16H), 2.11 (s, 3H, COC*H*$_3$), 2.04 ppm (s, 3H, COC*H*$_3$).; MALDI-TOF-MS: *m*/*z* calcd for $C_{20}H_{35}NNaO_{16}^+$: 568.185; found: 568.154.

1-5. NMR Spectra of Compounds **3α**, **3β**, **4α**, **4β**, **7**, **9**, and **10**

**Supplementary Figure 10, 11.**	^{1}H and ^{13}C NMR Spectra of compound **3α**
**Supplementary Figure 12, 13.**	^{1}H and ^{13}C NMR Spectra of compound **3β**
**Supplementary Figure 14.**	^{1}H Spectrum of compound **4α**
**Supplementary Figure 15.**	^{1}H Spectrum of compound **4β**
**Supplementary Figure 16, 17.**	^{1}H and ^{13}C NMR Spectra of compound **7**
**Supplementary Figure 18, 19.**	^{1}H and ^{13}C NMR Spectra of compound **9**
**Supplementary Figure 20.**	^{1}H Spectrum of compound **10**

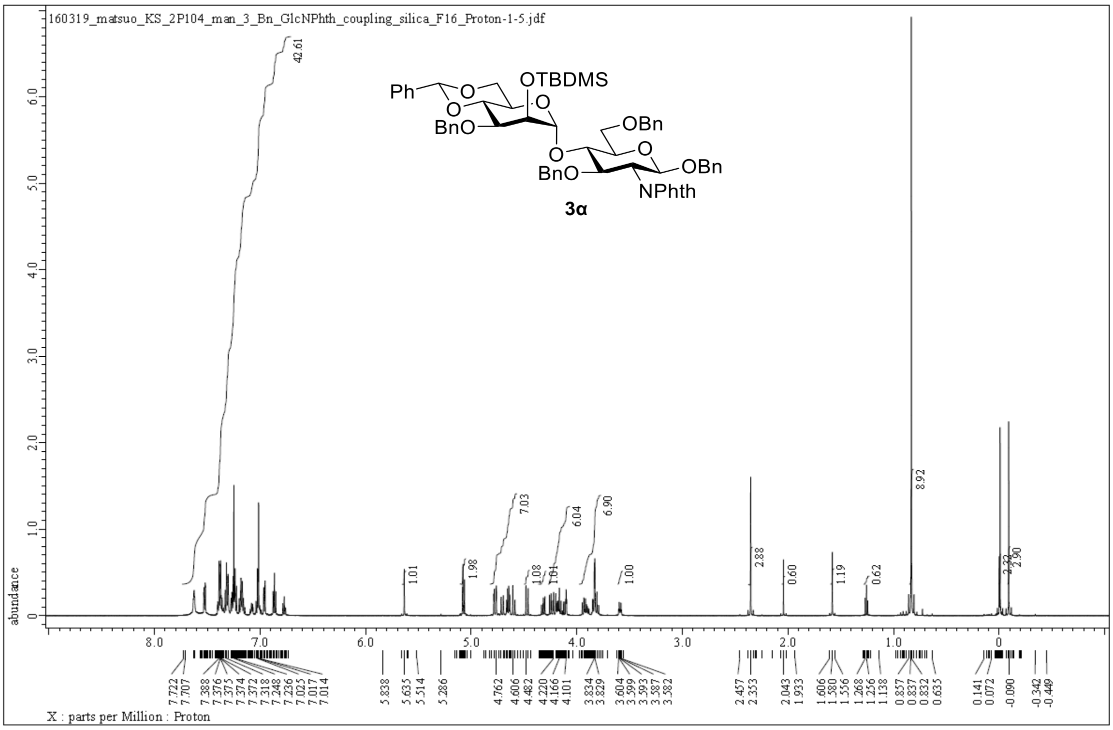

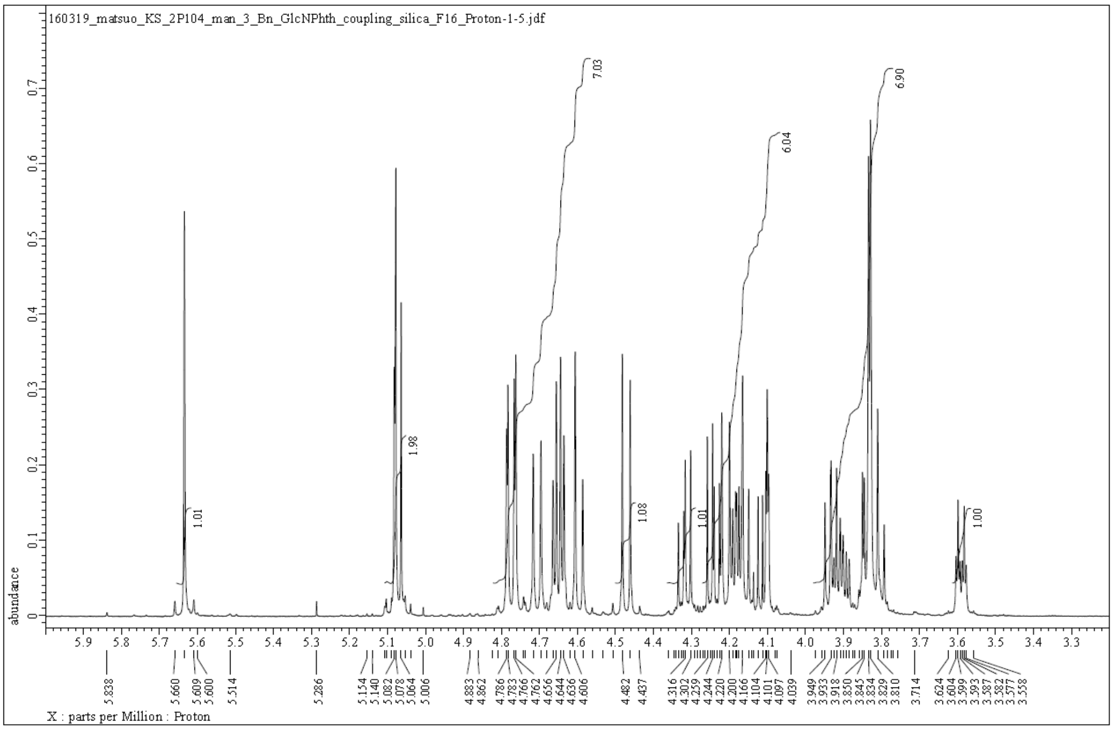

Supplementary Figure 10. ^{1}H NMR Spectrum of compound **3α**

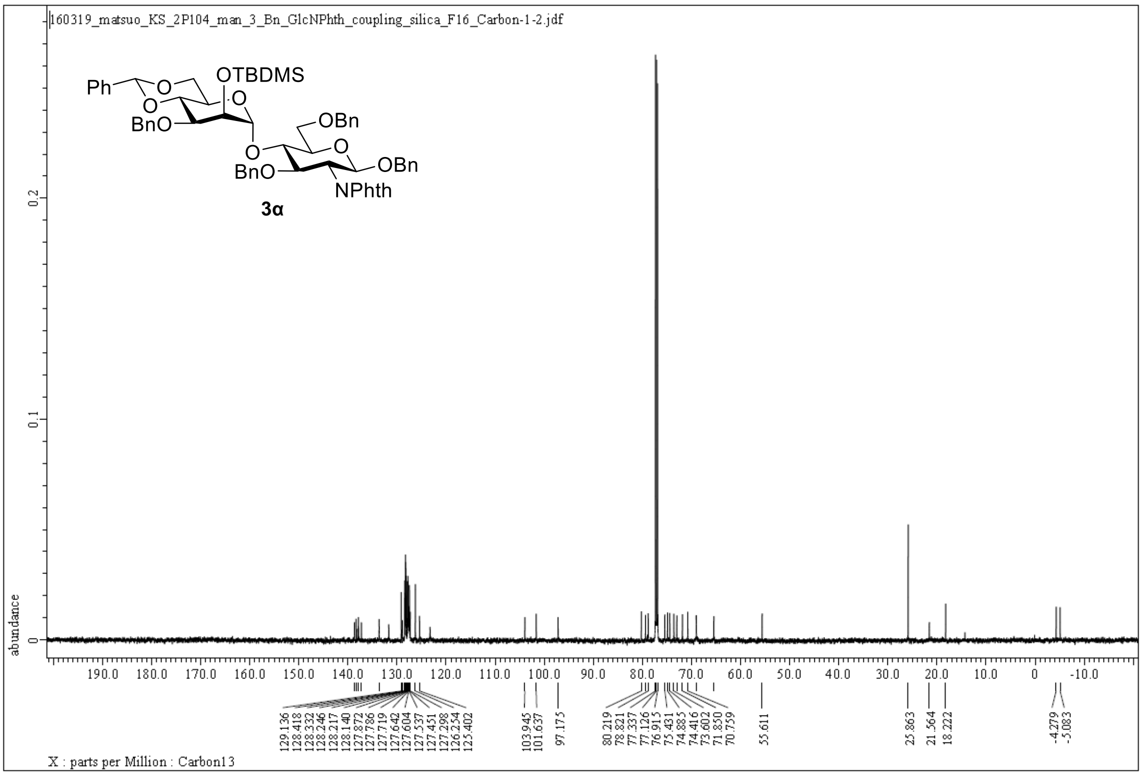

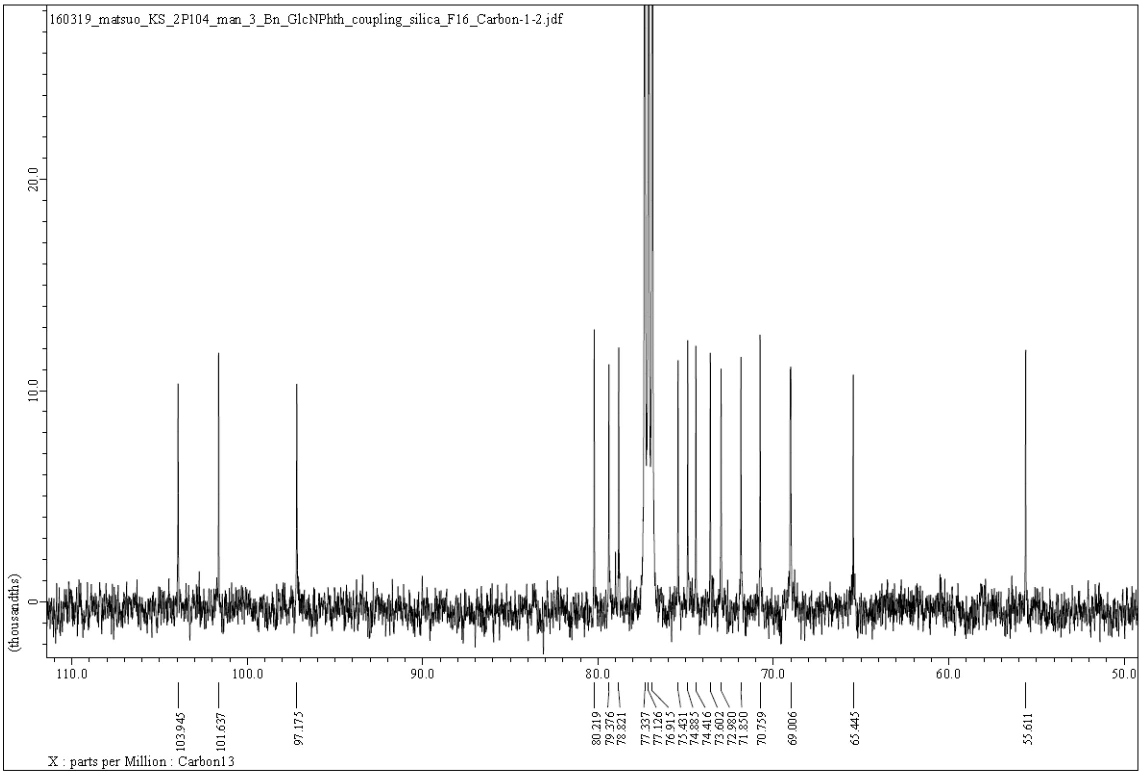

Supplementary Figure 11. ^{13}C NMR Spectrum of compound **3α**

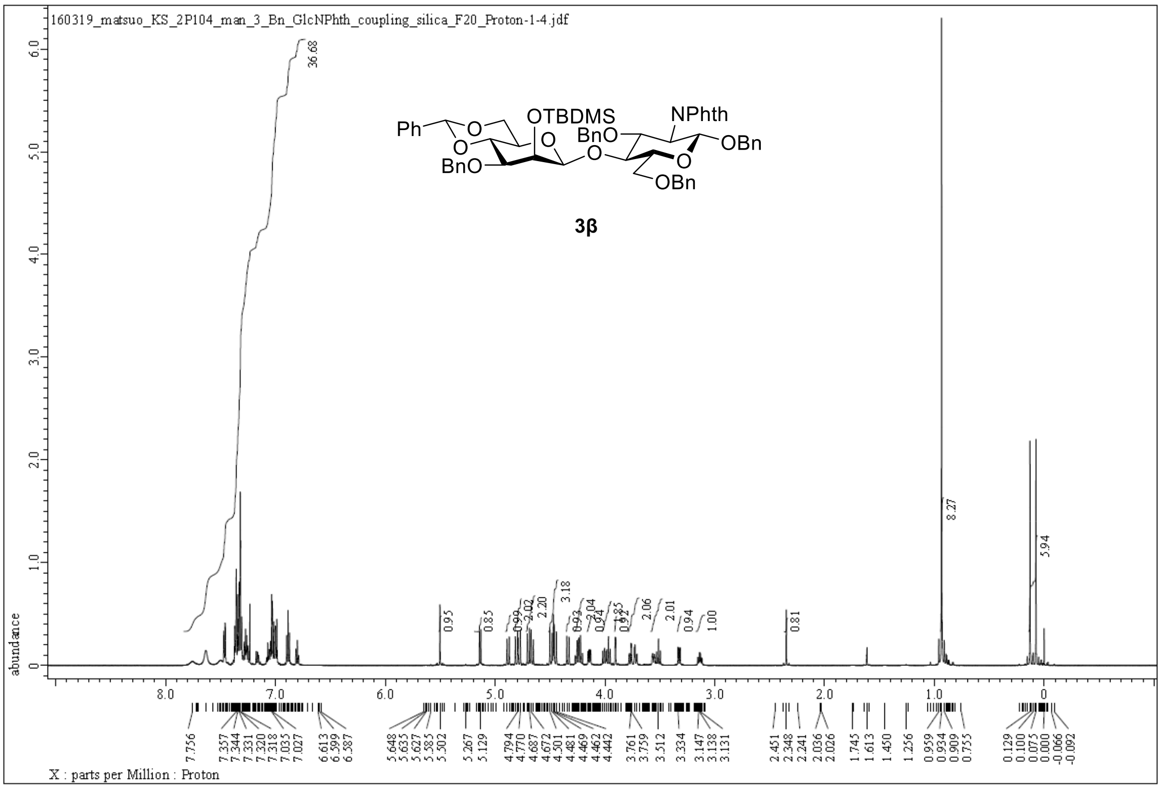

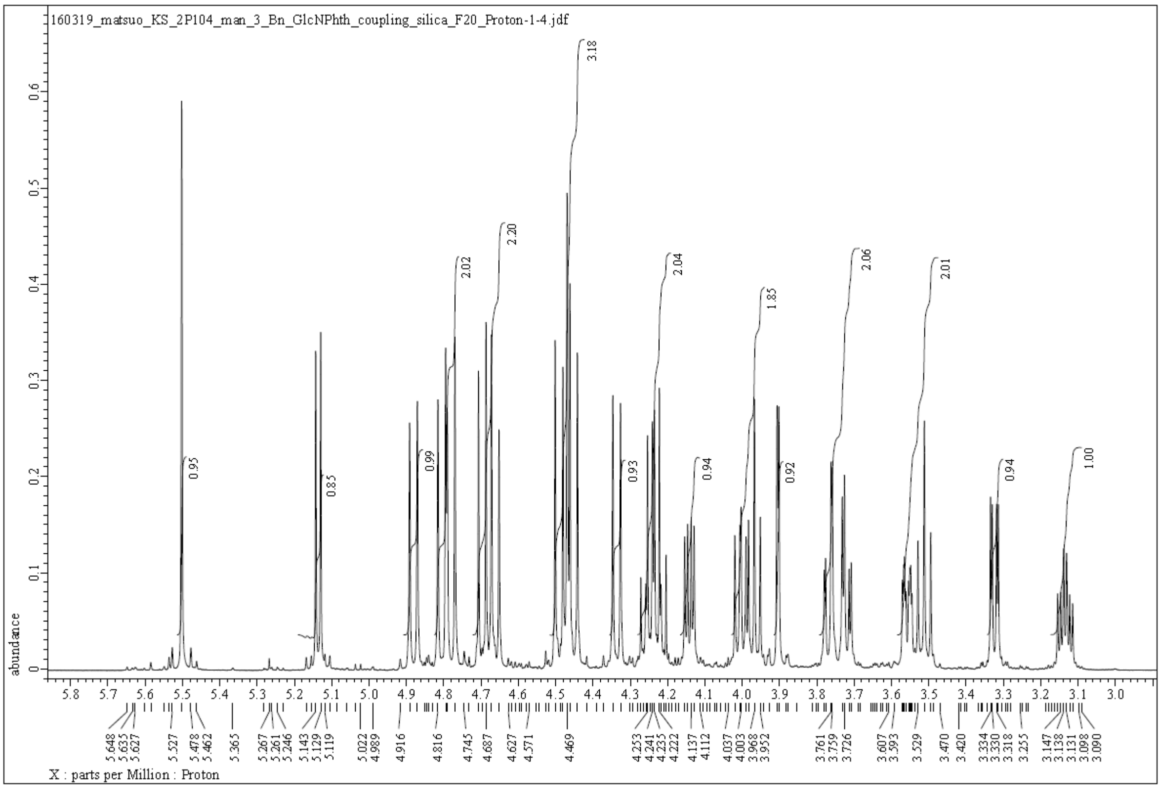

Supplementary Figure 12. ^{1}H NMR Spectrum of compound **3β**

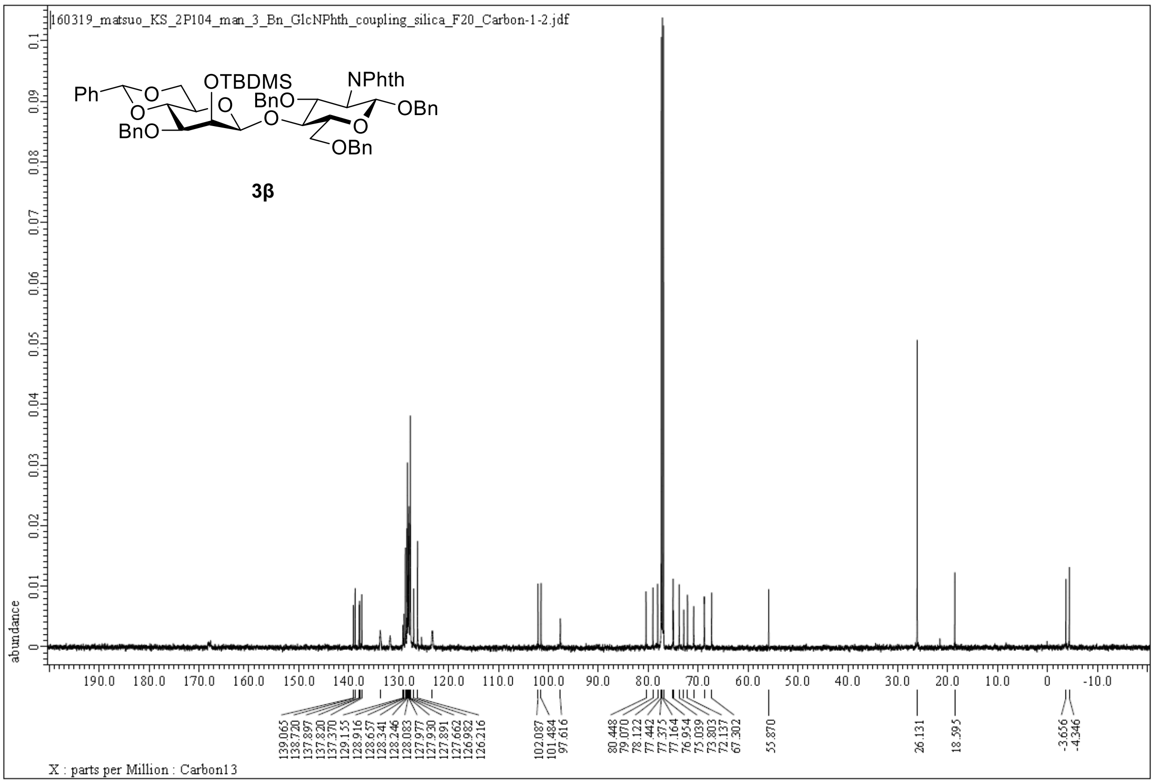

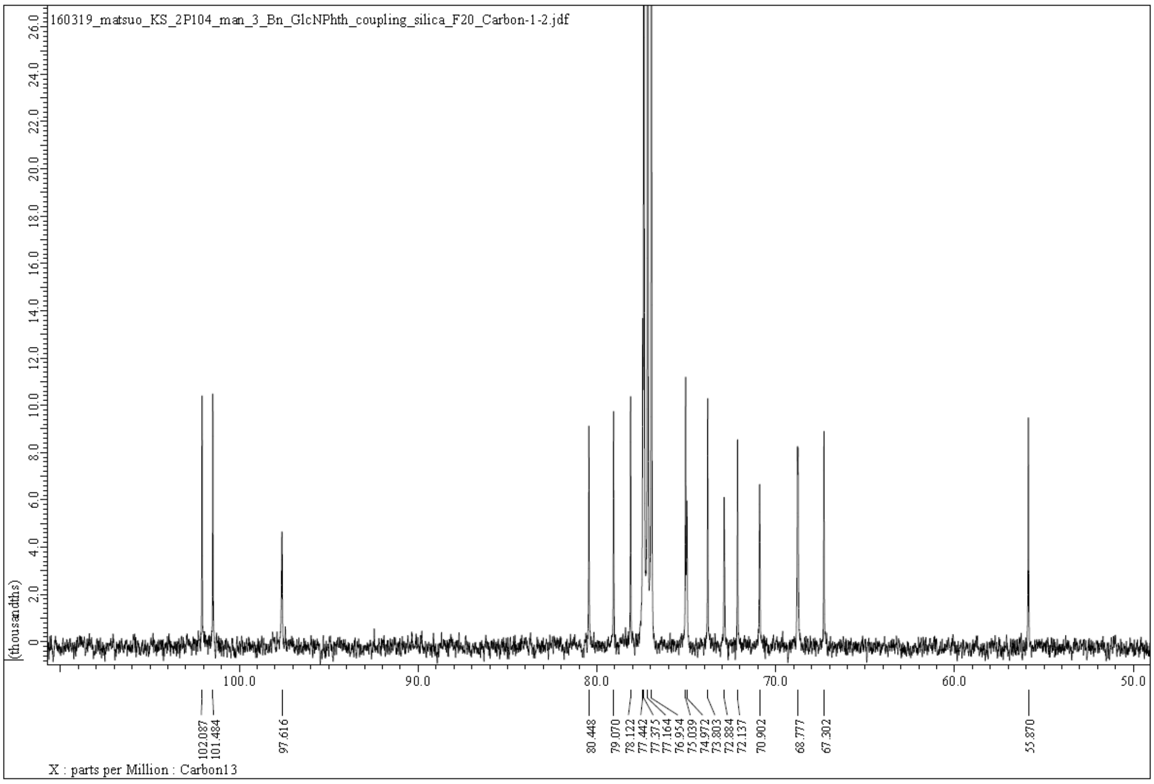

Supplementary Figure 13. ^{13}C NMR Spectrum of compound **3β**

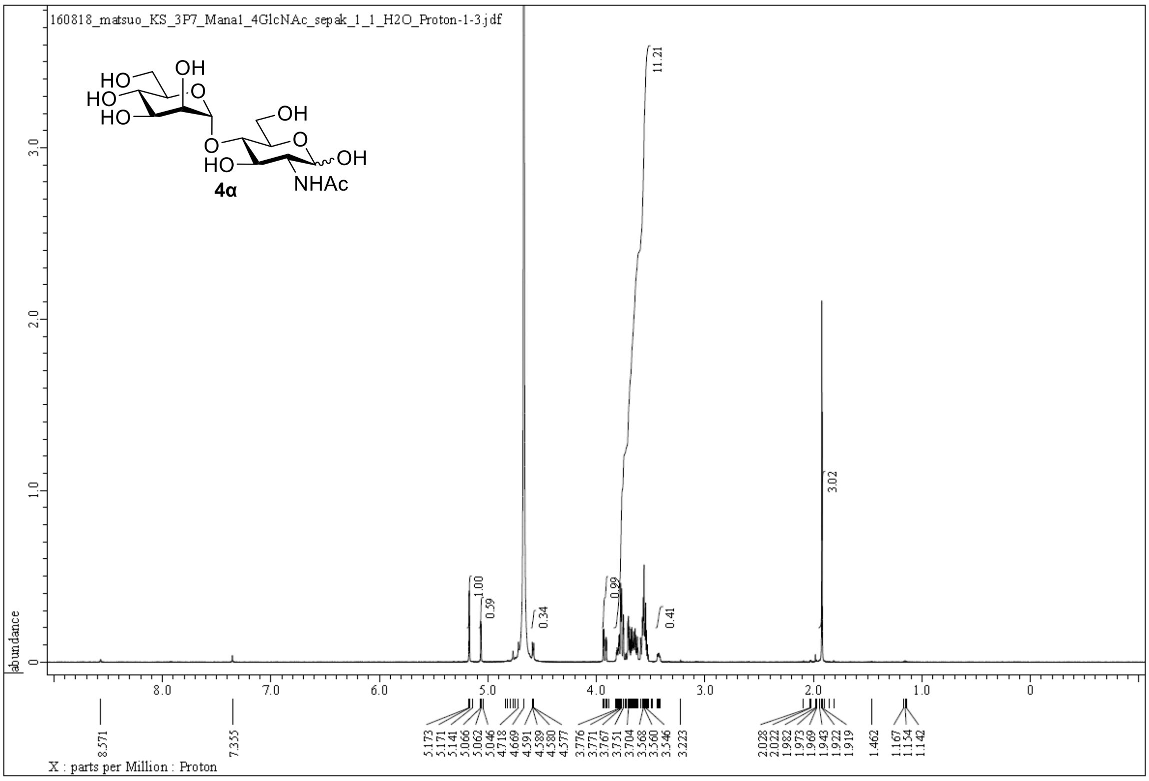

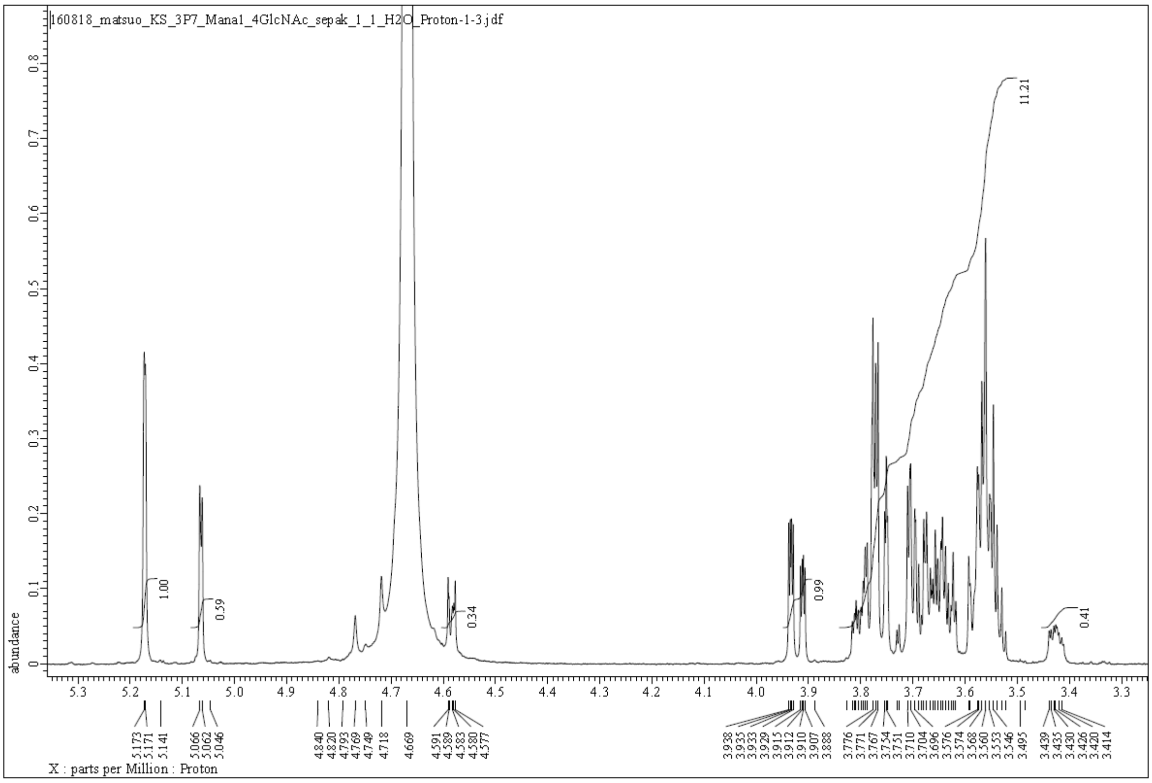

Supplementary Figure 14. [1]H Spectrum of compound **4α**

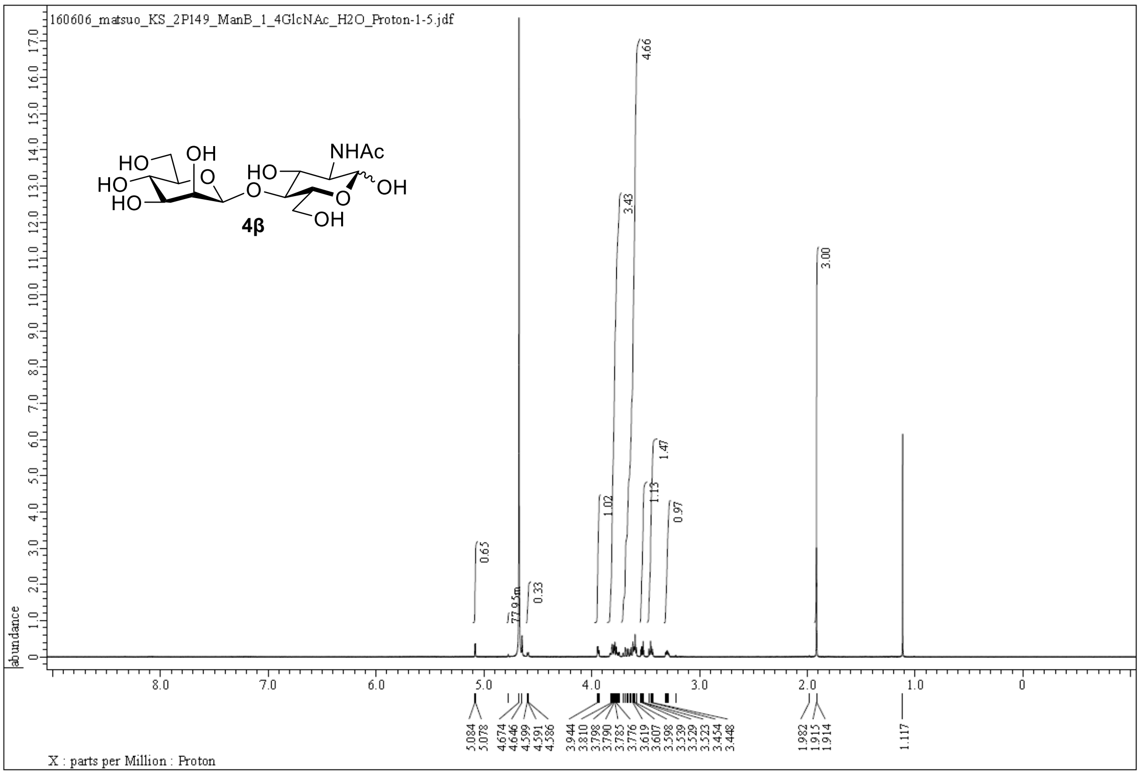

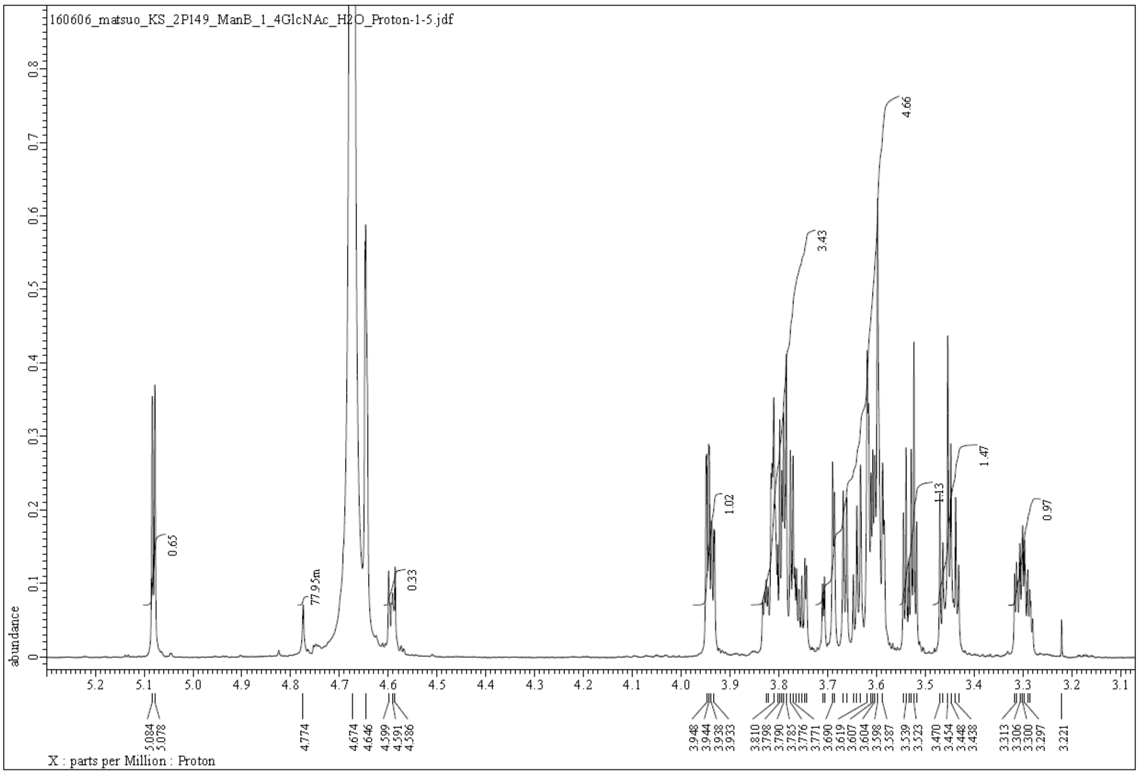

Supplementary Figure 15. ^{1}H Spectrum of compound **4α**

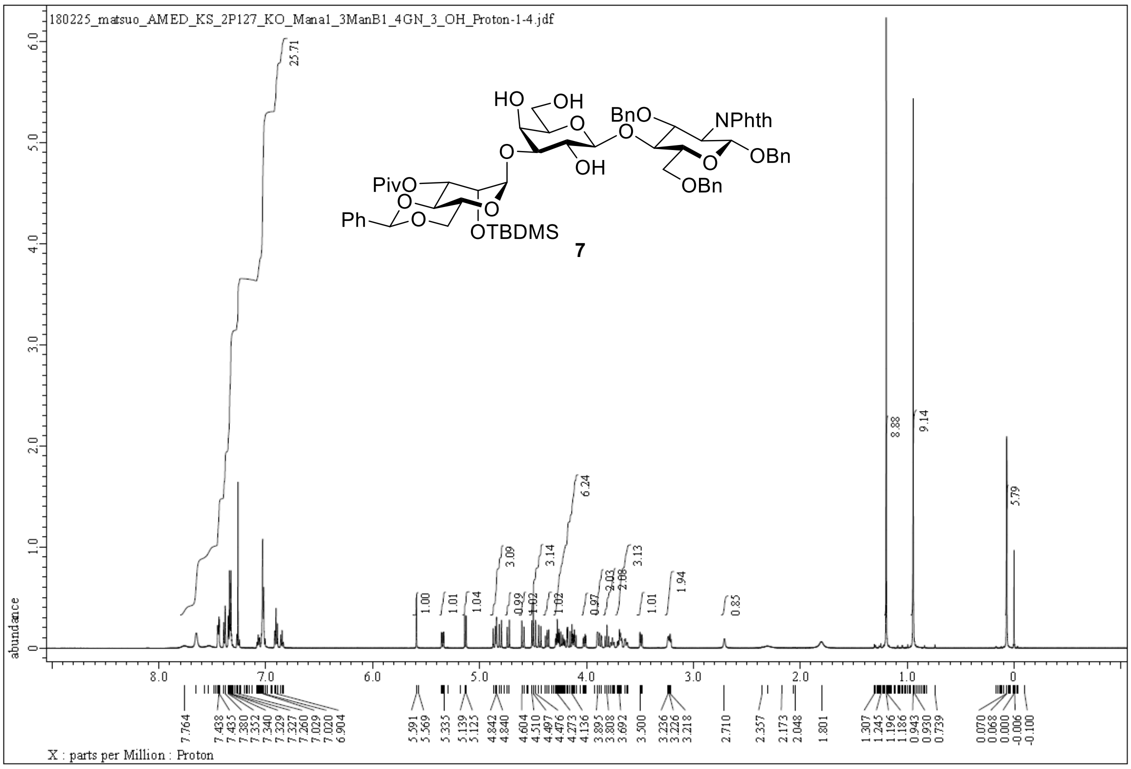

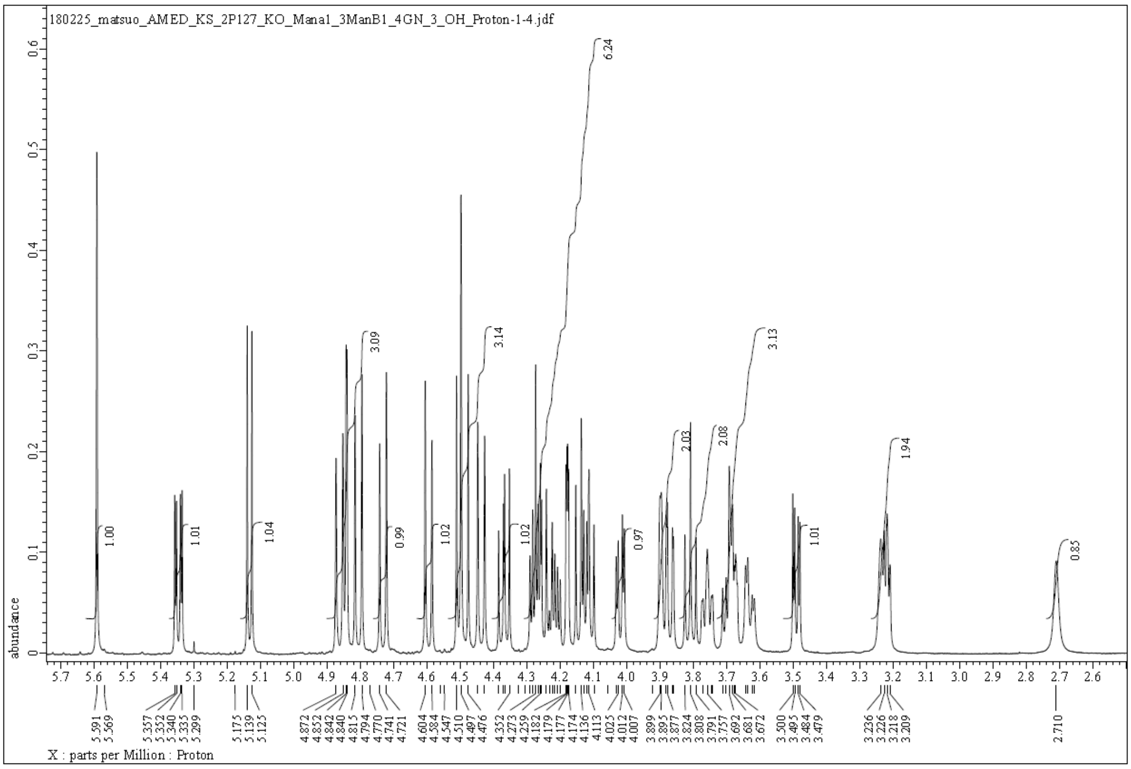

Supplementary Figure 16. [1]H Spectrum of compound **7**

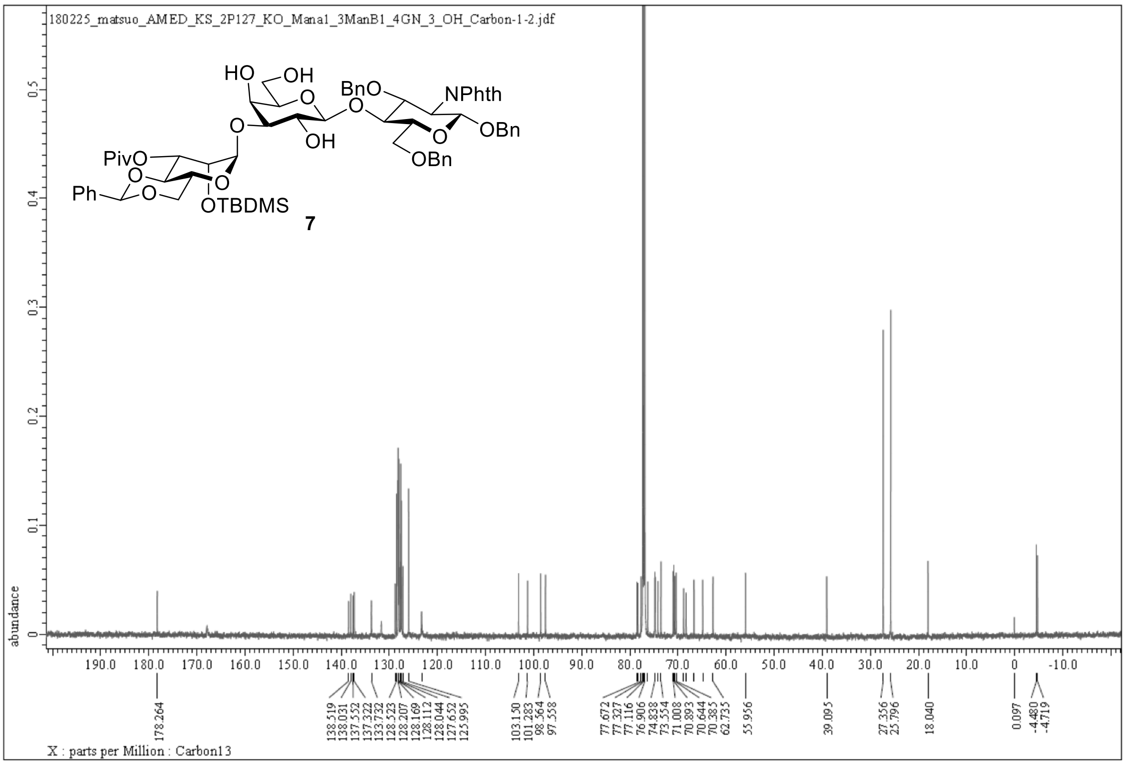

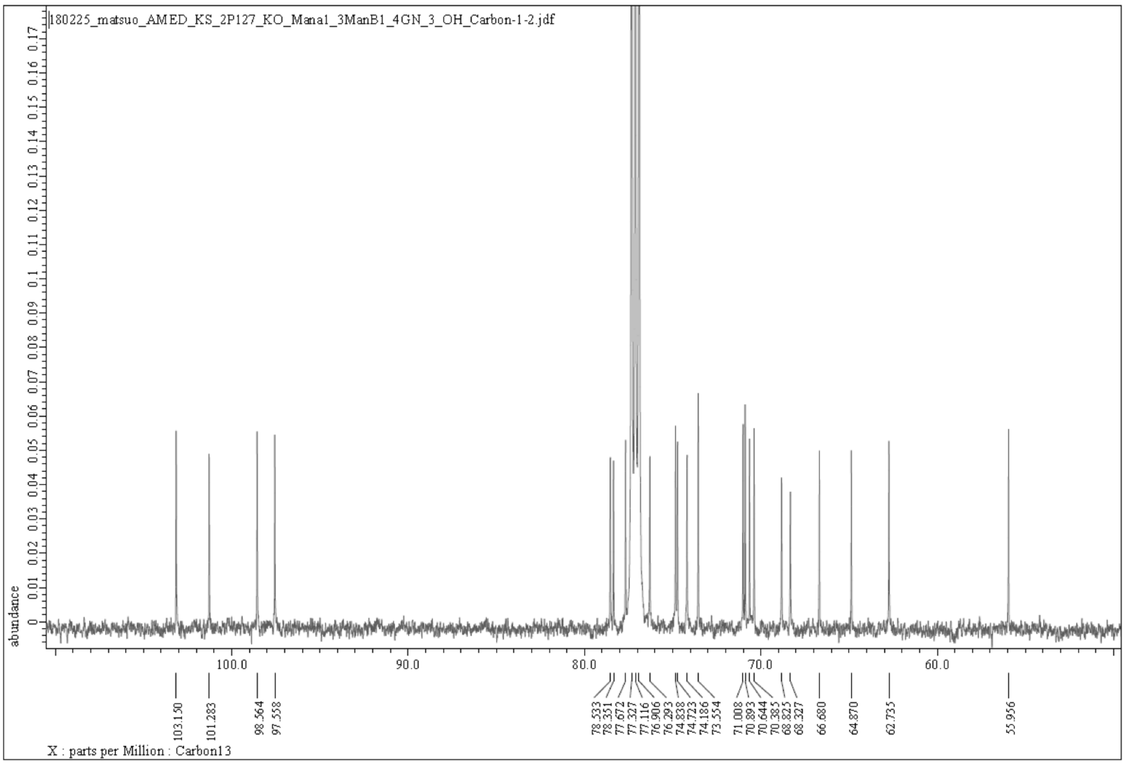

Supplementary Figure 17. ^{13}C NMR Spectrum of compound **7**

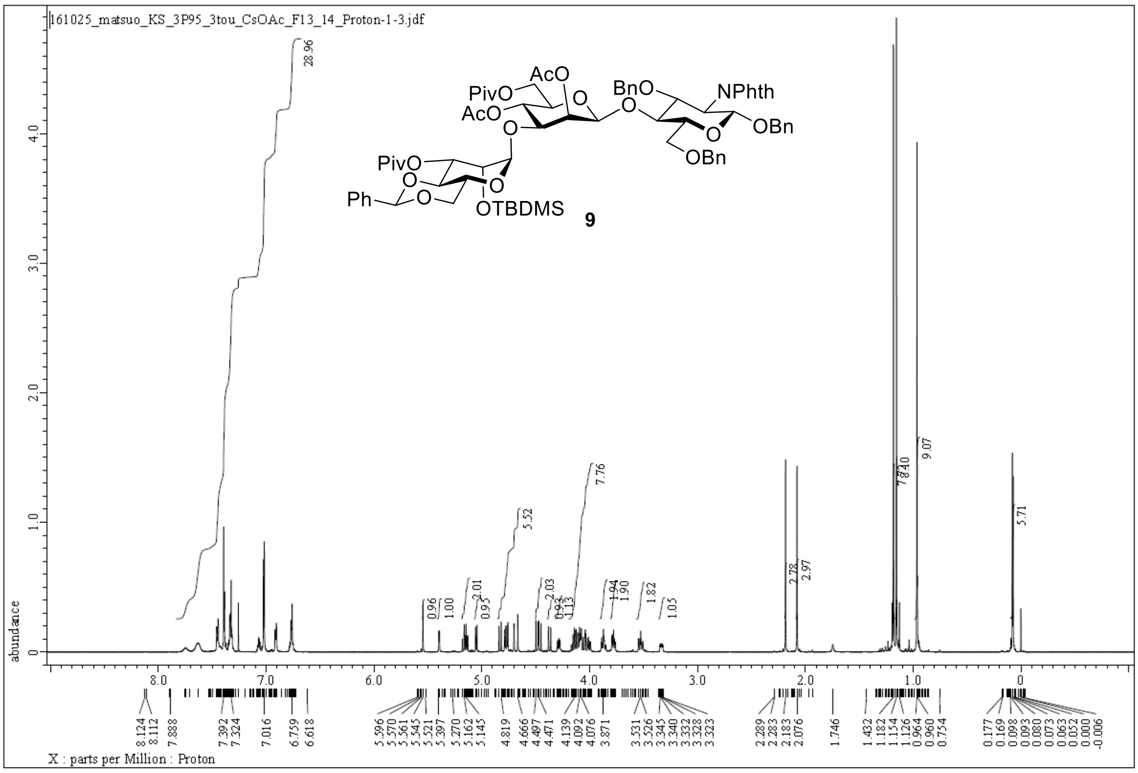

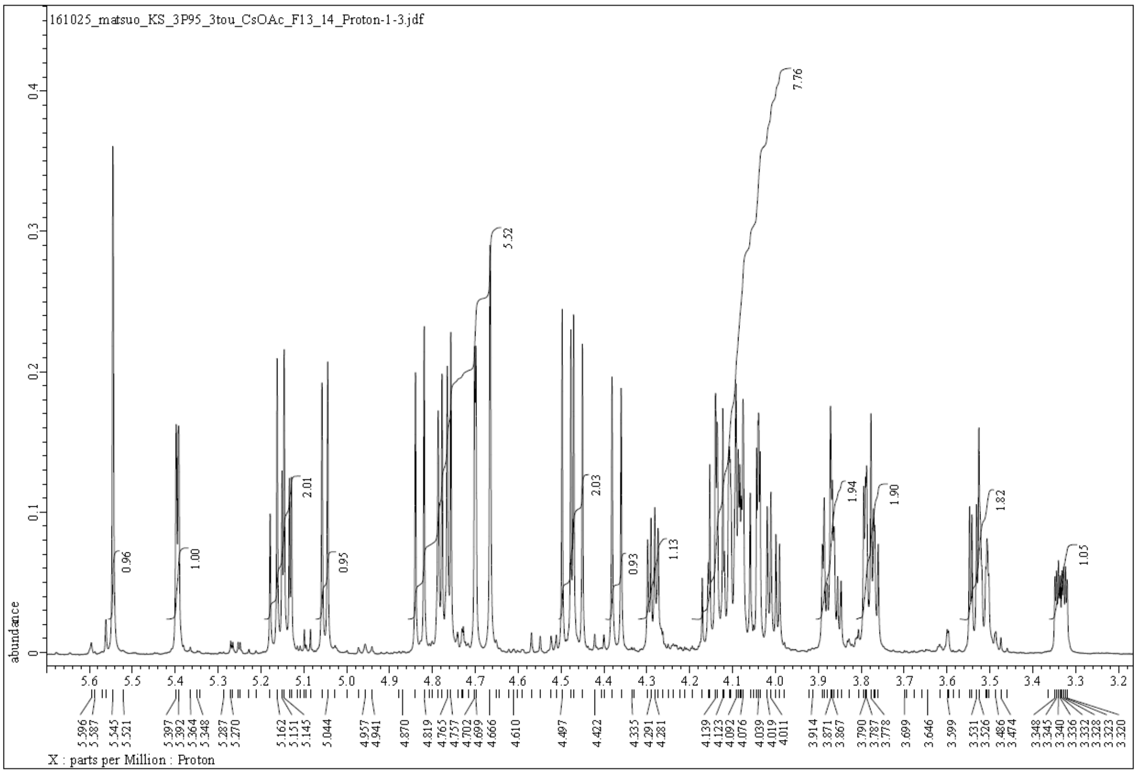

Supplementary Figure 18. ^{1}H Spectrum of compound **9**

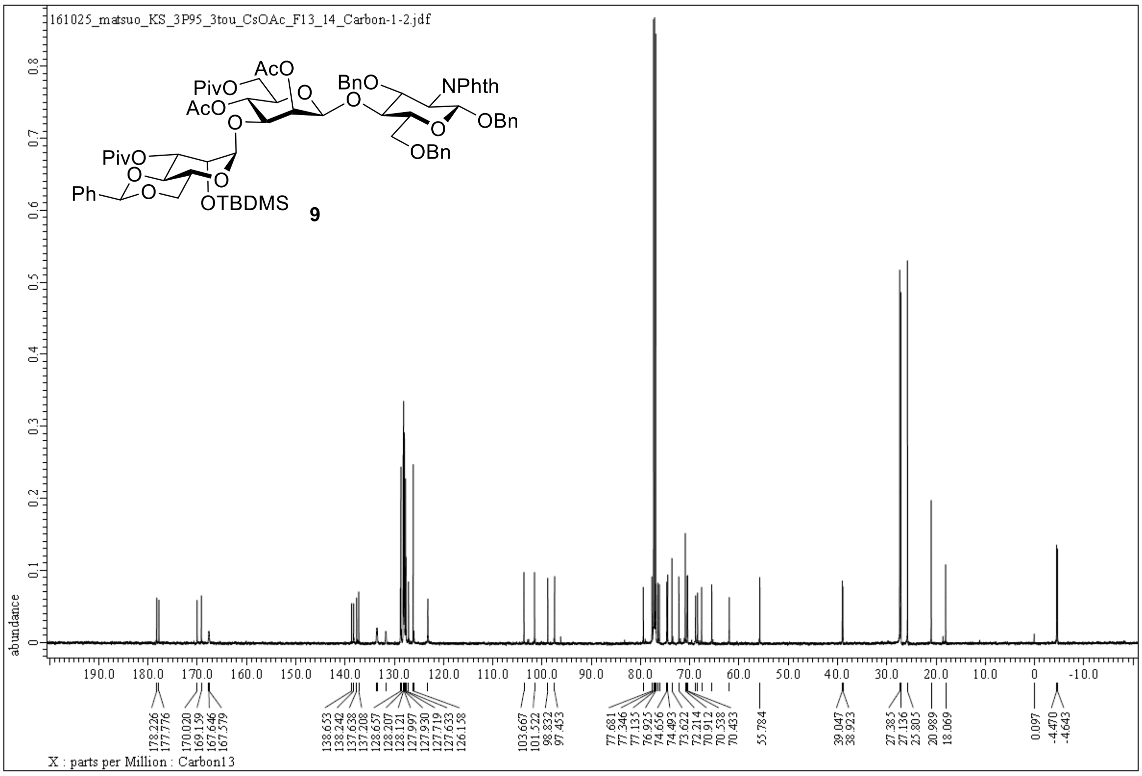

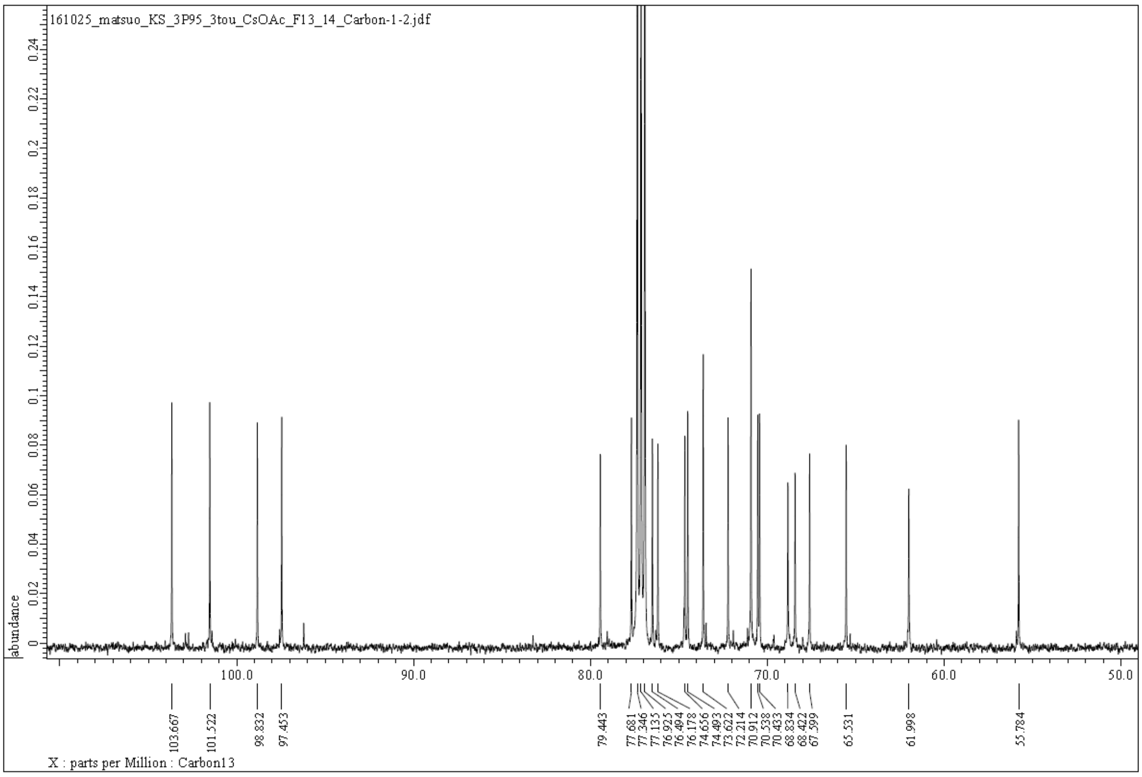

Supplementary Figure 19. ^{13}C NMR Spectrum of compound **9**

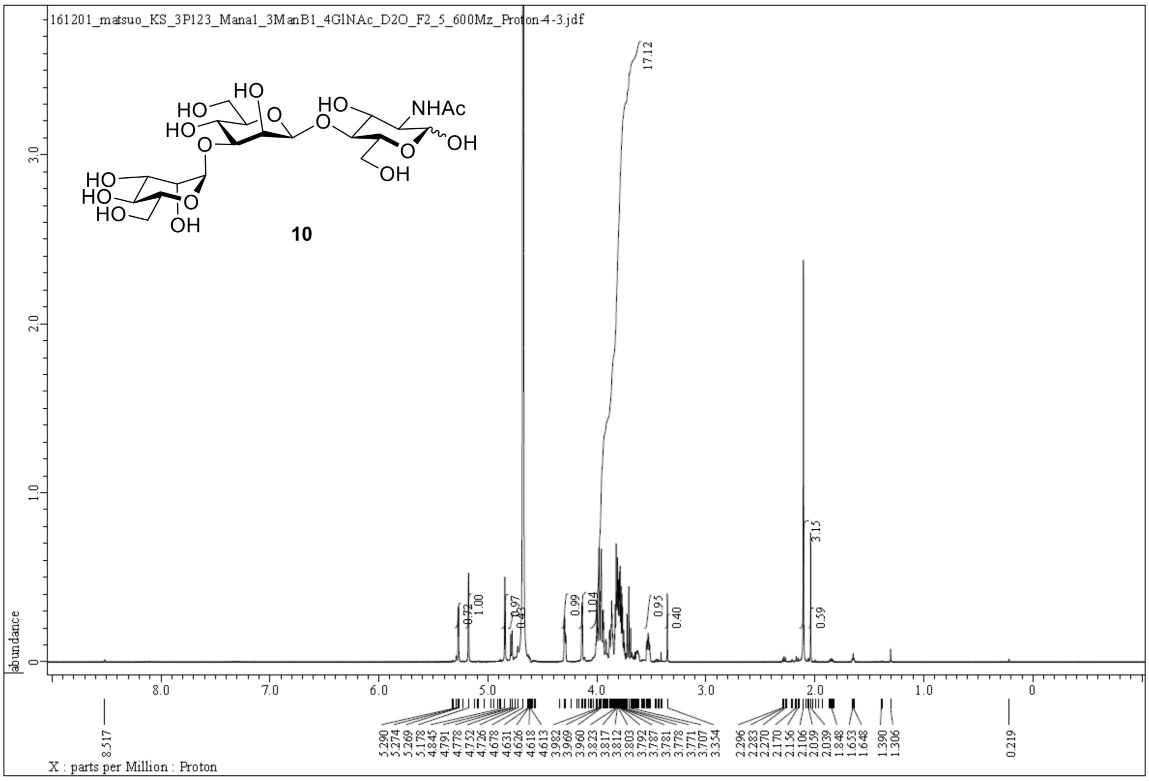

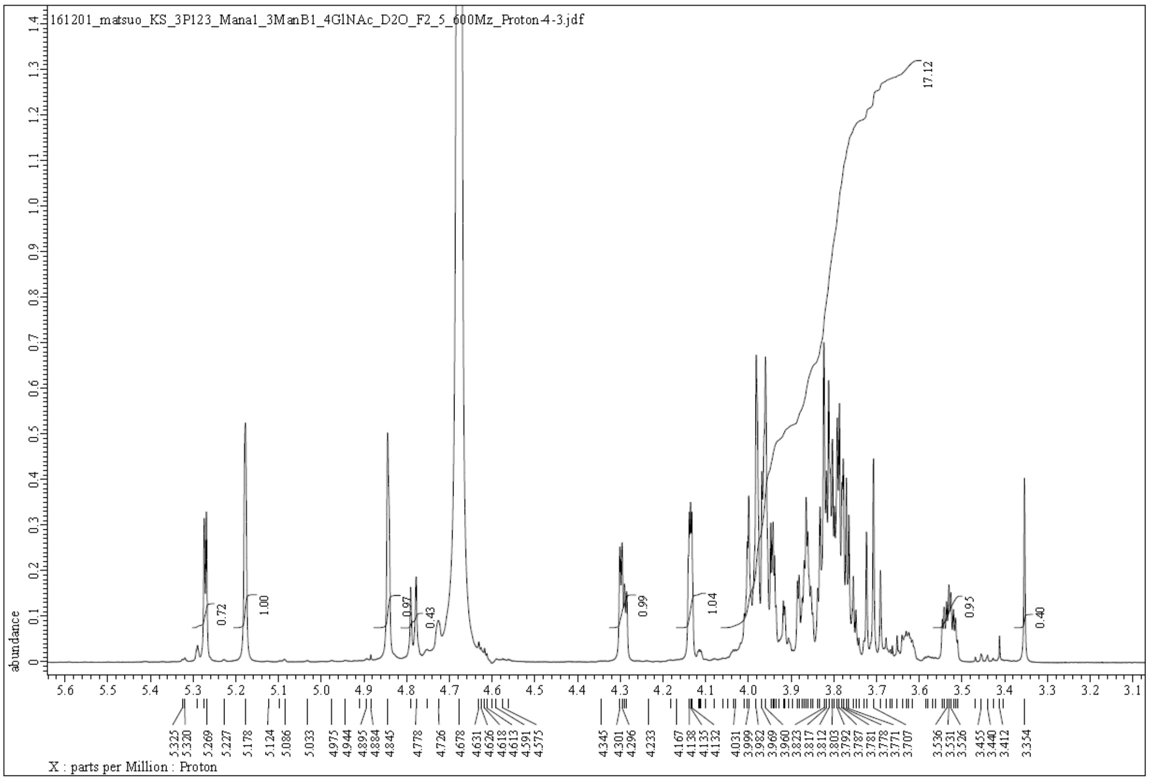

Supplementary Figure 20. ^{1}H NMR Spectrum of compound **10**
