# Supplementary material for: A bioactive mammalian disaccharide associated with autoimmunity activates STING-TBK1-dependent immune response
Source: Nat Commun. 2019 May 30;10:2377. doi: 10.1038/s41467-019-10319-5 (PMC6542856; doi:10.1038/s41467-019-10319-5)
Supplement: Supplementary file 2 — Reporting Summary [file 41467_2019_10319_MOESM2_ESM.pdf]

nature research

Corresponding author(s): Nan Yan

Last updated by author(s): Mar 27, 2019

Reporting Summary

Nature Research wishes to improve the reproducibility of the work that we publish. This form provides structure for consistency and transparency in reporting. For further information on Nature Research policies, see Authors & Referees and the Editorial Policy Checklist.

Statistics

For all statistical analyses, confirm that the following items are present in the figure legend, table legend, main text, or Methods section.

n/a	Confirmed	
☐	☒	The exact sample size (*n*) for each experimental group/condition, given as a discrete number and unit of measurement
☐	☒	A statement on whether measurements were taken from distinct samples or whether the same sample was measured repeatedly
☐	☒	The statistical test(s) used AND whether they are one- or two-sided<br>*Only common tests should be described solely by name; describe more complex techniques in the Methods section.*
☒	☐	A description of all covariates tested
☒	☐	A description of any assumptions or corrections, such as tests of normality and adjustment for multiple comparisons
☒	☐	A full description of the statistical parameters including central tendency (e.g. means) or other basic estimates (e.g. regression coefficient) AND variation (e.g. standard deviation) or associated estimates of uncertainty (e.g. confidence intervals)
☐	☒	For null hypothesis testing, the test statistic (e.g. *F*, *t*, *r*) with confidence intervals, effect sizes, degrees of freedom and *P* value noted<br>*Give P values as exact values whenever suitable.*
☒	☐	For Bayesian analysis, information on the choice of priors and Markov chain Monte Carlo settings
☒	☐	For hierarchical and complex designs, identification of the appropriate level for tests and full reporting of outcomes
☒	☐	Estimates of effect sizes (e.g. Cohen's *d*, Pearson's *r*), indicating how they were calculated

Our web collection on statistics for biologists contains articles on many of the points above.

Software and code

Policy information about availability of computer code

Data collection	Bio-Rad CFX96 qPCR instrument
Data analysis	Prism 7

For manuscripts utilizing custom algorithms or software that are central to the research but not yet described in published literature, software must be made available to editors/reviewers. We strongly encourage code deposition in a community repository (e.g. GitHub). See the Nature Research guidelines for submitting code & software for further information.

Data

Policy information about availability of data

All manuscripts must include a data availability statement. This statement should provide the following information, where applicable:

- Accession codes, unique identifiers, or web links for publicly available datasets
- A list of figures that have associated raw data
- A description of any restrictions on data availability

The data sets generated during and/or analysed during the current study are available from the corresponding author on reasonable request.

Field-specific reporting

Please select the one below that is the best fit for your research. If you are not sure, read the appropriate sections before making your selection.

☒ Life sciences ☐ Behavioural & social sciences ☐ Ecological, evolutionary & environmental sciences

For a reference copy of the document with all sections, see nature.com/documents/nr-reporting-summary-flat.pdf

Life sciences study design

All studies must disclose on these points even when the disclosure is negative.

Sample size	Sample size for each experiment is indicated in the figures or corresponding figure legends. The sample size of animal study was chosen based on previous experience in the lab.
Data exclusions	No data were excluded from the analysis.
Replication	All replicates reported in the manuscript are biological replicates.
Randomization	The age matched animals were randomized into control and drug-treated groups
Blinding	The researchers were blinded to allocation during analysis and outcome assessment. Animal experiments were performed in a blinded fashion when possible.

Reporting for specific materials, systems and methods

We require information from authors about some types of materials, experimental systems and methods used in many studies. Here, indicate whether each material, system or method listed is relevant to your study. If you are not sure if a list item applies to your research, read the appropriate section before selecting a response.

Materials & experimental systems

n/a	Involved in the study	
☐	☒	Antibodies
☐	☒	Eukaryotic cell lines
☒	☐	Palaeontology
☐	☒	Animals and other organisms
☒	☐	Human research participants
☒	☐	Clinical data

Methods

n/a	Involved in the study	
☒	☐	ChIP-seq
☒	☐	Flow cytometry
☒	☐	MRI-based neuroimaging

Antibodies

Antibodies used	Antibodies used in this study include anti-ubiquitin (rabbit, 1:1000 dilution, P4D1, Cell Signaling Technology), anti-Tubulin (mouse, 1:20,000 dilution, B-5-1-2, Sigma-Aldrich), anti-LC3 (rabbit, 1:1000 dilution,ab48394, Abcam), anti-STING (rabbit, 1:500 dilution, D2P2F, Cell Signaling), anti-GAPDH (rabbit, 1:1000 dilution, sc-25778, Santa Cruz), anti-Rab5 (rabbit, 1:1000 dilution,ab109534, Abcam),anti-HMGB1 and (rabbit; 1:2,000 dilution;ab18256; Abcam).
Validation	Antibodies are validated in previous published research in the lab.

Eukaryotic cell lines

Policy information about cell lines

Cell line source(s)	RAW264.7 cells were obtained from ATCC
Authentication	All cells were originally obtained from the ATCC cell repository
Mycoplasma contamination	All cell lines were tested for mycoplasma contamination by PCR genotyping
Commonly misidentified lines (See ICLAC register)	No commonly misidentified cell lines were used.

Animals and other organisms

Policy information about studies involving animals; ARRIVE guidelines recommended for reporting animal research

Laboratory animals	C57BL/6 mice
Wild animals	This study did not involve wild animals
Field-collected samples	The acquisition, care, housing, use, and disposition of animals in research were in compliance with applicable federal, state, and local, laws and regulations.

Ethics oversight	All mouse experiments were approved by ICUAC in UT Southwestern Medical Center.

Note that full information on the approval of the study protocol must also be provided in the manuscript.
